# Breast Cancer Survivors’ Perspectives on Motivational and Personalization Strategies in Mobile App–Based Physical Activity Coaching Interventions: Qualitative Study

**DOI:** 10.2196/18867

**Published:** 2020-09-21

**Authors:** Francisco Monteiro-Guerra, Gabriel Ruiz Signorelli, Octavio Rivera-Romero, Enrique Dorronzoro-Zubiete, Brian Caulfield

**Affiliations:** 1 Salumedia Tecnologías Seville Spain; 2 The Insight Centre for Data Analytics School of Public Health, Physiotherapy and Sports Science University College Dublin Dublin Ireland; 3 Oncoavanze Seville Spain; 4 Department of Electronic Technology Universidad de Sevilla Seville Spain

**Keywords:** mHealth, mobile app, mobile phone, coaching, physical activity, breast cancer

## Abstract

**Background:**

Despite growing evidence supporting the vital benefits of physical activity (PA) for breast cancer survivors, the majority do not meet the recommended levels of activity. Mobile app–based PA coaching interventions might be a feasible strategy to facilitate adherence of breast cancer survivors to the PA guidelines. To engage these individuals, PA apps need to be specifically designed based on their needs and preferences and to provide targeted support and motivation. However, more information is needed to understand how these technologies can provide individual and relevant experiences that have the ability to increase PA adherence and retain the individual’s interest in the long term.

**Objective:**

The aim of this study is to explore insights from breast cancer survivors on motivational and personalization strategies to be used in PA coaching apps and interventions.

**Methods:**

A qualitative study was conducted, using individual semistructured interviews, with 14 breast cancer survivors. The moderator asked open-ended questions and made use of a slideshow presentation to elicit the participants’ perspectives on potential mobile app–based intervention features. Transcribed interviews were evaluated by 3 reviewers using thematic content analysis.

**Results:**

Participants (mean age 53.3, SD 8.7 years) were White women. In total, 57% (8/14) of the participants did not adhere to the PA guidelines. In general, participants had access to and were interested in using technology. The identified themes included (1) barriers to PA, (2) psychological mediators of PA motivation, (3) needs and suggestions for reinforcing motivation support, (4) personalization aspects of the PA coaching experience, and (5) technology trustworthiness. Motivational determinants included perceived control, confidence and perceived growth, and connectedness. Participants were interested in having a straightforward app for monitoring and goal setting, which would include a prescribed activity program and schedule, and positive communication. Opinions varied in terms of social and game-like system possibilities. In addition, they expressed a desire for a highly personalized coaching experience based on as much information collected from them as possible (eg, disease stage, physical limitations, preferences) to provide individualized progress information, dynamic adjustment of the training plan, and context-aware activity suggestions (eg, based on weather and location). Participants also wanted the app to be validated or backed by professionals and were willing to share their data in exchange for a more personalized experience.

**Conclusions:**

This work suggests the need to develop simple, guiding, encouraging, trustworthy, and personalized PA coaching apps. The findings are in line with behavioral and personalization theories and methods that can be used to inform intervention design decisions. This paper opens new possibilities for the design of personalized and motivating PA coaching app experiences for breast cancer survivors, which might ultimately facilitate the sustained adherence of these individuals to the recommended levels of activity.

## Introduction

### Background

Breast cancer affects a large and growing population of women globally and is the second leading cause of death among women [1,2]. Fortunately, owing to advancements in screening and treatment, survival rates are on a steady rise [3]. In their journey during and after treatment, breast cancer survivors often need personalized support and encouragement to take a proactive approach in the self-management of their health and quality of life [4]. Participating in physical activity (PA), such as walking or jogging, is recommended as part of that process providing vital benefits to survivors, which include prevention of cancer recurrence, decrease in side effects from treatment, and improvements in fitness, body size, and quality of life [4-6]. However, most breast cancer survivors do not meet the PA guidelines and recommendations, with reported estimations of <10% in some studies based on self-reported PA data [7,8].

The growing demand for cost-effective alternatives to help people reach their personal activity targets, paired with the advancements in mobile monitoring technologies, has increased interest in the research and development of mobile PA coaching apps and interventions [9-11]. These are aimed at motivating people to change their activity behavior by means of coaching elements, such as measuring the user’s daily PA and providing feedback, guidance, and incentives, to generate awareness on current behavior and ultimately stimulate users to achieve and maintain an active lifestyle [12]. At present, these systems have the potential to provide engaging real time coaching experiences for users, and as suggested in related studies, these systems may be a feasible strategy for motivating cancer survivors to adhere to the recommended levels of PA [13,14].

Although there has been an increase in the popularity of PA apps, the vast majority is targeted at the healthy active population [15]. These apps are generally not suitable for individuals who may struggle with disease-specific barriers [16] and may have particular coaching needs for PA, such as breast cancer survivors [14]. To increase the likelihood of mobile PA coaching interventions being accepted and benefitting breast cancer survivors, it is essential to understand their contextual characteristics, needs, and preferences as end users of the technology to inform system design [17]. A few studies have looked into the specific requirements of breast cancer survivors for technology-based PA coaching interventions [14,18,19]. However, there is more to understand about what can hold their interest and motivate them to increase their PA levels in the long term. Previous work highlights the importance of exploring behavioral theories of motivation and personalization aspects in the design of these systems [20].

Using methods from behavioral theory in tools that aim to increase PA is believed to increase the success of these technology-supported interventions [21]. To achieve this, the technology should include evidence-based techniques drawn from behavior change theories that directly address the special barriers and motivators for PA adherence among the target users. Previous studies suggest that theories such as the social cognitive theory (SCT), transtheoretical model (TTM), and self-determination theory (SDT) should be used in the design of PA interventions for breast cancer survivors [22,23]; however, there is more to learn in this direction and to have a better understanding of the practical implications of technology implementation [23,24]. Furthermore, it is generally believed that tailoring or personalizing *any number of methods for creating communications individualized for their receivers* is associated with an increase in the effectiveness of behavior change systems [25,26]. Personalization can contribute to captivating and holding the user’s interest, which is of utmost importance in systems that generally struggle with user abandonment [27-29]. Recent work in this area deals specifically with real time personalization of PA coaching apps and suggests a number of strategies (eg, user targeting, goal setting, adaptation) that can be used and combined for that purpose [12,20]. In line with this, previous studies with breast cancer survivors seem to suggest a preference for an app experience that is highly tailored to each user [14,24,30]. However, there is still very little evidence on which factors can be taken into account for the creation of individualized experiences for these individuals.

### Objectives

To our knowledge, no study has thoroughly explored the perspectives of breast cancer survivors toward personalization and motivational aspects in PA coaching apps. Therefore, we conducted a qualitative research study with the goal of obtaining practical insights from breast cancer survivors on how a PA app may be accepted and provide personally relevant and engaging experiences to these individuals. These insights were applied to established constructs from theory on behavior change, SCT, and SDT and on personalized coaching to inform future app design.

## Methods

### Study Design

This study adopts a qualitative approach comprising semistructured interviews. The methods used were based, in part, on the qualitative work of Robertson et al [13] on mobile health (mHealth) PA intervention preferences in cancer survivors.

Individual face-to-face semistructured interview sessions were conducted to gather information on the patients’ experiences and perspectives about PA coaching apps, including their thoughts and experiences (eg, barriers and facilitators or motivators) about PA and the use of PA apps, and their insights on various coaching strategies and characteristics.

### Theoretical Basis

To understand the determinants of PA among breast cancer survivors, we considered 2 theoretical approaches that address this issue of motivation: the SCT and the SDT. The SDT by Ryan and Deci [31] suggests that self-motivation evolves from the degree to which a person’s innate psychological or basic needs are met within their social context. The 3 psychological needs identified in the theory are competence, a person’s ability to interact effectively within their environment; autonomy, a person’s perceived control of their choices; and relatedness, sense of connectedness to others in the immediate environment [32]. The theory proposes that satisfying these needs may promote and facilitate motivation by developing more intrinsic motivations—performing tasks for inherent enjoyment, interest, and pleasure of accomplishing them—consequently leading to task persistence [31]. The SCT, as proposed by Bandura et al [33,34], establishes that a person with given beliefs, information, and needs functioning in given social and physical environments will engage in a behavior that will have a consequent outcome. The 2 primary determinants of behavior in SCT are expected outcomes: expectations about the outcomes resulting from engaging in behavior and self-efficacy, or efficacy, expectations (beliefs) about one’s ability to engage in or execute the behavior. Self-efficacy is widely recognized as one of the strongest determinants of PA participation [35]. Another core component of SCT is the sociostructural factor, which includes impediments and facilitators to performing the desired behavior.

Another aspect that has been associated with an increase in engagement and effectiveness of behavior change systems is personalization, which may help increase the intended effects of the communication provided by these technologies. In particular, the model of real time personalization in PA coaching systems [12] defines 7 different personalization concepts that can be explored in these systems to provide unique and dynamic experiences to each user. They are feedback, presenting individuals with descriptive, comparative, and/or evaluative information about themselves; goal setting, presenting the user with short-term and long-term goals that can create a feeling of progress; user targeting, conveying, explicitly or implicitly, that the communication is designed specifically for the user; interhuman interaction, support for any form of interaction with other real human beings; adaptation, direct communication to individuals’ status on key theoretical determinants of the behavior of interest; context awareness, providing relevant information and/or services to the user based on the user’s context (not including user characteristics) and needs or goals; and self-learning, the ability to update the internal model of the user over time by recording and learning from the various user interactions with the app.

Finally, gamification, *the use of game design elements in nongame contexts* [36], was also considered in the analysis of this study. Gamification has been broadly used in health and fitness apps, with studies suggesting its potential to create fun and engaging experiences for the users [37,38]. Gamification methods include points, rewards, competition, avatars, and themes.

### Recruitment

The inclusion criteria for research participants were oncology patients with a history of breast cancer (stages I-III) that finished primary curative treatment (ie, surgery, radiotherapy, and chemotherapy), aged>18 years, own and use a mobile phone or smartphone, and have the ability to read and speak Spanish, with no known impairments or comorbidities, and no restrictions on PA.

The participants were recruited from the Oncology Clinic of the Oncoavanze group (in Seville, Spain) by placing a phone call to the eligible individuals identified in the Oncoavanze patient database. Recruitment was conducted until saturation of results was reached, which was considered when there was no new information (themes) arising from the qualitative data.

This study was approved by the Biomedical Research and Ethics Committee of the Junta de Andalucia in Spain. Subjects’ agreement for participation was obtained through an informed consent process.

All individuals who decided to participate had attended the interview sessions, and none of them dropped out of the study.

### Data Collection

The study sessions took place in a private room at the Oncoavanze headquarters, where only the session facilitator and the participant were present. Data collection ran from July 12 to 27, 2018, and was conducted by the main investigator (FG), a biomedical engineer and PhD candidate trained in qualitative research methods. The sessions lasted 35-60 min.

#### Standardized Questionnaires and Demographics

At the beginning of the sessions, initial assessments were made to describe the participants’ characteristics. Structured questionnaires and scales were administered to collect data on demographics (questionnaire), satisfaction with life scale (SWLS; Spanish version of the SWLS [39]), technology use and interest (questionnaire based on a study by Robertson et al [13]), and PA (questionnaire: Spanish version of the International Physical Activity Questionnaire-Short Form, IPAQ-SF [40]). The IPAQ-SF results were also used to extrapolate the adherence of participants to the PA guidelines [41], a step done by GS.

#### Semistructured Interviews

During the interviews, FG provided trigger questions to the participants based on a semistructured interview guide and took field notes. The first part of the interview was a discussion in which the moderator asked open-ended questions, followed by conversational probes as appropriate. This part included questions about participants’ experiences with managing their health and PA, including barriers and facilitators, previous use of mobile apps, and perspectives toward a tailored PA app and related requirements (Multimedia Appendix 1). During the second part, after a general introduction about PA apps and personalization, the moderator used a slideshow presentation of possible app features (Multimedia Appendix 2) and asked participants to share their thoughts and opinions on the featured content. In an attempt to avoid obtaining insights biased by the example features shown, before showing each of them, the moderator asked participants if they could contextualize the strategy being discussed with a real life example not necessarily related to PA or apps (eg, can you think of an example in your life where defining goals was important or useful to you?).

Two researchers, FG and OR, designed the interview guide following an iterative process (Multimedia Appendix 1). The questions and slideshow presentation were derived from the work of Robertson et al [13] and chosen to combine concepts of personalization in PA coaching apps [12,20] and behavioral theory [31,34] (eg, monitoring, goal setting, social connectedness, and context awareness) as well as practical questions (eg, participant perspectives and preferences). Given that the literature suggests that breast cancer survivors’ main and preferred form of exercise is walking, including activities of daily living [18,24], and to have a more comprehensive research in that direction, the features presented targeted specifically such types of aerobic activities. In addition, the slideshow presentation and explanations were designed assuming an audience with low technical knowledge.

Interviews were conducted in Spanish. All sessions were recorded with a digital audio recorder and transcribed verbatim. All transcribed data were deidentified and securely stored on password-protected computers.

Participants’ quotes considered in the analysis were later translated to English for writing the Results section. It was not a literal translation owing to the amount of unique linguistic expressions used by the participants, which are characteristic of their region. Translated transcripts were validated linguistically and culturally through back-translation techniques and evaluated by bilingual translators.

### Data Analysis

Data analysis was conducted by 3 independent reviewers (FG, OR, and ED) on the original Spanish transcripts, and consisted of both deductive and inductive approaches. FG is a PhD student trained in qualitative research methods, and both OR and ED are senior researchers with experience in qualitative research.

A preliminary coding frame was defined by 2 researchers (FG and OR) based on the main topics of interest assessed using the interview guide: barriers and facilitators on PA, insights on previous app use, perspectives on personalization, and opinions on potential app functionalities and characteristics. This initial deductive phase allowed the coders to become familiar with the content and filter out irrelevant information.

After this initial step, the 2 coders (OR and ED) used an approach drawn from inductive methods, based on a thematic content analysis [42], and theoretically informed deductive methods. In the latter method, SCT [33,35,43] and SDT [31,44] constructs, personalized coaching strategies [12,20,25], and gamification mechanics [37] helped to identify internal and external motivational influences on participants’ PA adherence and PA app use. The analysis involved a comparison of the aforementioned theories with the raw data. This comparison enabled the researchers to code theoretically relevant constructs (eg, competence, relatedness) that then helped to identify themes associated with motivational determinants of PA adherence (eg, confidence and perceived growth, social connectedness), intervention characteristics (eg, activity monitoring and goal setting, interacting with other users), and how they can support motivation by influencing the motivational determinants.

At the end of each iteration, the themes and subthemes were reviewed and refined. Recurring themes and subthemes were consolidated and coded through an iterative process.

Preliminary findings were then reviewed by a third coder (FG), who reviewed it against the interview transcripts and field notes and proposed a final readjustment to the results. Any discrepancies during the coding process were resolved by consensus. Illustrative quotes for each subtheme were selected from the data set.

Microsoft Excel version 16 was used to organize content within thematic categories.

## Results

### Participant Characteristics

Semistructured interviews were conducted with 14 patients with breast cancer. The age of the participants ranged from 43 to 69 years, with a mean of 52.8 (SD 8.8) years and a median of 50 (IQR 47.3-56.8) years. The number of years since diagnosis ranged from 1 to 11.5, with a mean of 4.25 (SD 2.8) years and a median of 3.9 (IQR 2.5-5.4) years. Participants were well educated and most were employed (11/14, 79%). The IPAQ-SF scores indicated that most participants had a moderate or high level of PA (12/14, 86%). When analyzing the IPAQ-SF answers in terms of the activity type and intensity, 8 participants reported only doing low-intensity activities (eg, light walks) and therefore did not adhere to the PA guidelines. In addition, 7 of the 14 participants reported spending 5 hours a day of sedentary behavior. The mean SWLS score was 17.6 (SD 4.2), indicating a neutral-to-good satisfaction with life. Most participants reported being very interested in technology, reported having ready access to technological devices, and have shown high usage of a variety of technology functionalities (Multimedia Appendix 3). However, some reported neutral self-reported skill with technology (5/14, 36%). More details are provided in Table 1.

**Table 1:** Participant characteristics (N=14).

Characteristics		Values, n (%)
**Marital status**		
	Single	4 (29)
	Married	10 (71)
	Divorced	0 (0)
**Education**		
	Basic school	1 (7)
	High school	2 (14)
	Higher education	2 (14)
	University or college	9 (64)
**Current employment status**		
	Not working	3 (21)
	Employed	11 (79)
Receiving pharmacological treatment		10 (71)
Indication for PA^a^		11 (79)
**IPAQ-SF^b^ level**		
	High	1 (7)
	Moderate	11 (79)
	Low	2 (14)
Adheres to PA guidelines (>150 min per week=moderate activity or >75 min per week=vigorous activity)^c^		6 (43)
**Interest in technology**		
	Agree or strongly agree	12 (86)
	Neutral	2 (14)
	Disagree or strongly disagree	0 (0)
**Self-reported skill with technology**		
	Agree or strongly agree	9 (64)
	Neutral	5 (36)
	Disagree or strongly disagree	0 (0)
“**I like to experiment with new technology”**		
	Agree or strongly agree	7 (50)
	Neutral	5 (36)
	Disagree or strongly disagree	2 (14)

^a^PA: physical activity.

^b^IPAQ-SF: International Physical Activity Questionnaire-Short Form.

^c^On the basis of the IPAQ-SF answers.

### Themes

**Figure 1:**
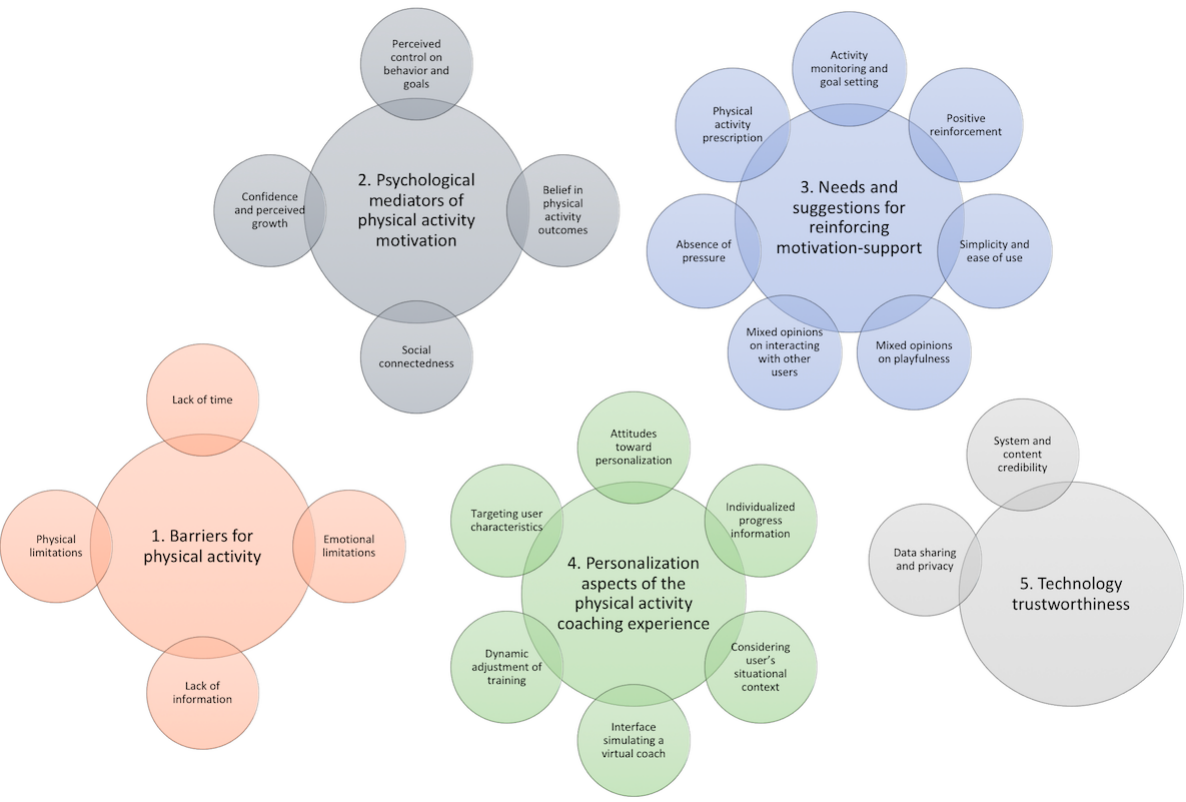
Schematic representation of themes and subthemes.

The following themes were identified from the interview data. These include aspects related to PA adherence and design considerations for PA apps in breast cancer survivorship. The main themes identified were as follows: (1) barriers to PA, (2) psychological mediators of PA motivation, (3) needs and suggestions for reinforcing motivation support, (4) personalization aspects of the PA coaching experience, and (5) technology trustworthiness (Figure 1). Subthemes are introduced below within each of the main themes’ sections. A table with supporting quotation is provided in Multimedia Appendix 4.

#### Barriers for PA

Although participants seemed generally aware of the importance of PA, they often expressed the challenges and difficulties that prevent them from being more active. These included lack of time, physical limitations, emotional challenges, and lack of information. From the SCT perspective, these can be defined as sociostructural factors that can inhibit the performance of the behavior.

##### Lack of Time

Time management challenges were often raised across most participants. The 2 main reasons associated with these challenges were work schedule and being in charge of relatives:

My daughters are still ten years old, and during the morning I work, I take them to school and I pick them up from school. So, when I don’t have to take them to school, I walk [to work]. […]. At work, I am sitting most of the morning. And, in the afternoon, I combine their extracurricular activities [in own schedule], so I’m not very constant in maintaining physical activity.

P03, aged 48 years, moderate PA level, high skill with technology

We live so fast that we don’t know how to manage time, and [then] we say we don’t have time to do it. […] The barrier is time management because I’m in an important professional moment […] and on top of that I’m also a mum, so I choose [to leave aside] sports… I leave it [sports] as a last priority […].

P10, aged 39 years, moderate PA level, neutral skill with technology

##### Physical Limitations

Many breast cancer survivors report experiencing side effects from the disease and treatment, particularly physical problems, which can limit the extent to which they participate in PA. Among the physical burdens reported by the participants were lymphedema, weight gain, changes in body image, muscular and joint pain, fatigue, and neuropathy:

Because you know that due to the “remains” of the neuropathy, fatigue, tiredness, and muscular pains, either you move or everything [all the physical burdens] will be much worse... it will hurt more or you will be more tired.

P02, aged 50 years, moderate PA level, neutral skill with technology

[I felt] many barriers associated with the lymphedema and weight [gained], when doing activity [exercise] with gym equipment.

P05, aged 43 years, moderate PA level, high skill with technology

[...] between what they put in you [referring to medication] and the volume of weight that you gain [...] you get out of breath.

P08, aged 47 years, moderate PA level, high skill with technology

##### Emotional Challenges

Some participants suggested that their emotions were also affected after treatment and that it could negatively influence their motivation to participate in PA. Such emotional instability was reflected in feeling more stressed and depressed. They often suggested how common it was to have bad thoughts after the cancer:

We are people who stress easily and manage stress even worse now than before the disease. Also because of the medication we receive... it gives some emotional instability…

P02, aged 50 years, moderate PA level, neutral skill with technology

For a while now I have stopped doing it [physical activity] because I was a little depressed and stressed.

P04, aged 48 years, moderate PA level, high skill with technology

People start [saying] “Oh, I’m feeling bad, I’m feeling bad, I’m feeling bad...”, and in that way, you don’t recover.

P04, aged 48 years, moderate PA level, high skill with technology

##### Lack of Information

Although most participants were advised to be more active by their health care professionals, they reported having unmet information needs on the type, amount, and duration of PA. It was also suggested that some doctors were outdated and recommended rest instead of PA:

[...] the order of exercises or of stretching [...] or, maybe, if something hurts, what exercises can be better for you, or what type of things you shouldn’t do.

P02, aged 50 years, moderate PA level, neutral skill with technology

What makes me angry is that there are many doctors that are still very outdated and the first thing they tell you is to rest… that’s the easiest… and then people “rust”.

P04, aged 48 years, moderate PA level, high skill with technology

[...] you often find yourself a bit disoriented [...]. [Talking about mastectomy] I found out much later, once I had already finished the treatment, about lymphedema… I did not know that I had to do some exercises.

P07, aged 56 years, moderate PA level, high skill with technology

#### Psychological Mediators of PA Motivation

This theme presents the psychological mediators of behavior change associated with breast cancer survivors’ adherence to PA. These were analyzed through the lenses of the SDT and SCT approaches and included perceived control on behavior and goals, confidence and perceived growth, belief in PA outcomes, and social connectedness.

##### Perceived Control of Behavior and Goals

Perceived control is related to the concept of autonomy, as defined in the SDT, which is associated with the feeling of being the origin of one’s own behaviors and experiencing volition in action. Participants reported a willingness to play an active role in managing their health as it could positively influence their own and their loved ones’ lives. This can be associated with a sense of ownership and value alignment, which influence perceived autonomy:

It was me [the source of motivation]. Me, because I had to save my children… I was pregnant.

P04, aged 48 years, moderate PA level, high skill with technology

I have been given an opportunity of being here, in life, again... and I have to make the most out of it, doing good for others and being happy.

P11, aged 69 years, moderate PA level, high skill with technology

In addition, there seemed to be a need to self-regulate and feel in control of their PA experience. Participants highlighted several aspects related to acting in alignment with their own goals and choosing a personal PA strategy, which are characteristics of autonomy support:

The objective is to always have some group or activity to sign up to, always… be it pilates or aquagym. [...] not leaving it to when I feel like doing it, but to present myself with a concrete activity. The specific objective is general maintenance, controlling… I do it for my health [...]

P01, aged 57 years, low PA level, neutral skill with technology

[...] Now I’m more interested in the subject of calories, due to the pill that I’m taking, anti-hormone, and all that. I want to have more control over it. [...] Setting goals and objectives is always important. [...]

P13, aged 47 years, moderate PA level, high skill with technology

I think that in the end it’s you who sets your own targets and if you feel good with what you are doing then that’s all you need.

P07, aged 56 years, moderate PA level, high skill with technology

##### Confidence and Perceived Growth

A person's confidence in one’s own capacity to perform a behavior or a task is defined, according to SCT, as self-efficacy. It is often associated with the concept of competence of SDT, which is about the need for perceived capability and growth in performing the desired behavior. In the context of breast cancer survivors, the belief in their capacity to perform more PA seemed to be limited by their physical and psychological barriers and fears. Participants referred to their difficulties in starting, or restarting, to perform PA after treatment and how they needed to start slow. There was this general perception that they had lower physical capabilities than before treatment:

I went back to training and you want to start almost where you left off, and physically you are not in the same conditions, then it costs you a little, it's like starting from scratch.

P05, aged 43 years, moderate PA level, high skill with technology

In the beginning, when I finished the treatments, it was very difficult to do physical activity. […] I had more fear than actual [self-] confidence […].

P02, aged 50 years, moderate PA level, neutral skill with technology

Participants talked about the importance of feeling improvements, for example being progressively challenged, being successful in achieving defined goals, and feeling improvements on their biological processes (eg, weight loss and heart rate). This is related to the concepts of growth and mastery of experience, from SCT and SDT, which involve exercising one’s capacities, acquiring new skills, or receiving constructive feedback. These, over time, are believed to increase confidence, autonomy, and satisfaction:

So, you realize how you are improving, because, of course throughout the time that you are burning calories, you are losing weight and you are improving your performance.

P04, aged 48 years, moderate PA level, high skill with technology

[That] these objectives would vary if you have been accomplishing part of them… that they would be each time more complex and allowing you to overcome yourself. So, in this way, […] you start feeling better because every time you can achieve more objectives, doing more stuff or goals.

P02, aged 50 years, moderate PA level, neutral skill with technology

Some participants also suggested that their source of confidence was associated with their success in overcoming their disease or relapses. In this line, having positive and constructive thoughts was an important part of their process of acceptance and self-motivation after cancer. This is related to the idea of positive self-talk in SCT, which is believed to increase self-efficacy (confidence):

I think it motivated me psychologically that I could say “I did it [recovered] and I was capable of doing it”, and it was extremely complicated… [it was] a pretty hard road [process of recovery].

P05, aged 43 years, moderate PA level, high skill with technology

##### Belief in PA Outcomes

Interviewees seemed to be aware of the importance of integrating PA in their lives. This can be associated with the concept of outcome expectations from SCT. Participants’ awareness of the physical and psychological benefits of PA in breast cancer survivorship was highlighted as a source of motivation to be more active:

The fact that you can move more, helps you relax your mind. The fact that you can find yourself more agile, helps you to feel better about yourself… [...] I believe sport is fundamental [...], it’s about constant improvement… which, for people who come out of cancer [...], and that have been in a capsule of medicines, pain and mental focus [...], when that capsule opens you are so broken that any reasonable target is seven or ten steps that you climb… sport helps a lot in that sense.

P02, aged 50 years, moderate PA level, neutral skill with technology

It motivates me that I feel better [by being active]… I feel much better physically and psychologically. More lively, as if with more strength, energy… yes, that’s what motivates me.

P05, aged 43 years, moderate PA level, high skill with technology

##### Social Connectedness

Social connectedness, or relatedness, is one of the psychological needs of SDT. Aspects related to social support by health care professionals, peers, and close ones were raised during the interviews. Participants had varied opinions on relatedness depending on the source of support. These aspects are also related to the component of SCT on sociostructural factors that can affect behavior.

###### Health Care Professionals

Opinions on interactions with health care professionals who took part in their treatment were generally very positive. Often, participants stated that they were crucial in helping them become motivated to cope with the disease and to adhere to PA, and particularly highlighted the importance of the psycho-oncologists:

The psychologist is very important because you talk to her [...], she cheers you up and then you feel like coming back and do stuff [talking about PA].

P05, aged 43 years, moderate PA level, high skill with technology

[After treatment] I had more fear than actual [self-] confidence. I was lucky to go to a psycho-oncologist [...] and she encouraged me not to stop doing physical activity… I also met [name of an exercise trainer] [...] which was a very important help.

P02, aged 50 years, moderate PA level, neutral skill with technology

###### Peers

Opinions on peer support and interaction were not straightforward, with a mixture of positive and negative thoughts by participants. On the positive side, because breast cancer survivors share this common life experience, they can understand each other’s situations, feelings, and fears, which enhances the feeling of relatedness and facilitates positive interactions between them:

If you get together with someone that has a similar experience, and you talk and share your feelings, that’s a support.

P01, aged 57 years, low PA level, neutral skill with technology

Especially when you are with people who are going through the same as you, then you vent a lot [...] sometimes you don’t have to talk, just hanging out, laughing, and disconnecting from problems... it helps a lot.

P05, aged 43 years, moderate PA level, high skill with technology

Positive opinions regarding performing PA in groups were also expressed by participants. In particular, it was suggested that it could help in socializing and avoiding getting bored. In addition, the fact that they could see others like themselves performing PA seemed to be motivating:

Doing PA in a group is different… but if you are doing alone, you do not progress, and sometimes you give up because you get bored. When you do not progress, it is interesting if someone motivates you in some way.

P05, aged 43 years, moderate PA level, high skill with technology

I think that another very funny thing about activities is to be able to socialize with other people.

P07, aged 56 years, moderate PA level, high skill with technology

Seeing how [the trainer] trained other women and see how she made them swim and do exercises… to me, that was important, truly.

P02, aged 50 years, moderate PA level, neutral skill with technology

Negative feelings toward peer interactions were based on the potential negative impact that it could have on their own emotions. Some participants reported an almost unavoidable tendency to speak about their cancer experiences, often negative and sad, in peer groups, which could prevent them from potentially benefiting from being part of these groups. In some cases, this was accentuated by their health professionals who recommended them to avoid such interactions at certain stages of the treatment. In addition, group heterogeneity in terms of PA levels was also suggested by participants as a barrier to adherence:

[People of the group] were talking about the disease, the types of intervention, etc.: “This doctor is not good” and then it turns out they were talking about your doctor; “This type of intervention no” and it turns out it was yours [...]. [Name of the oncologist] once told me “don’t interact with anyone... interact with people who don’t have cancer.”

P03, aged 48 years, moderate PA level, high skill with technology

But, in that case [talking about a Nordic Walking group], it was my own [physical] limit [as the barrier]... because I was in one homogeneous group [with higher PA level] and I was asphyxiated [couldn’t handle the activity level].

P03, aged 48 years, moderate PA level, high skill with technology

###### Family and Friends

Related ones had a particularly relevant role in providing support for participants to better manage their health, being identified as an important source of motivation. Some participants reported a very positive influence on their families’ involvement in their PA experiences:

I try to share these goals with the people around me so that they know [my goals] and they can help me, as a support network.

P02, aged 50 years, moderate PA level, neutral skill with technology

#### Needs and Suggestions for Reinforcing Motivation Support

This theme identifies a number of strategies and characteristics that, from the participant’s perspective, could address their motivational needs for PA: activity monitoring and goal setting, PA prescription, positive reinforcement, absence of pressure, simplicity and ease of use, mixed opinions on playfulness, and mixed opinions on interacting with other users.

##### Activity Monitoring and Goal Setting

Receiving information on the activity performed, together with goal setting, may help monitor the desired behavior and take actions to regulate it. Both monitoring and regulating are the main components of self-control, which is a construct used in SCT-based interventions. The participants were very interested in being able to monitor the different aspects related to their activities (including steps, duration, distance, and speed). In addition, some were particularly interested in knowing the calories burnt during exercise:

All the information [regarding own PA] seems important to me… the more information you have on an exercise that you are doing, the better. Everything seems important because sometimes you are guided [by this information].

P05, aged 43 years, moderate PA level, high skill with technology

I find it super motivating… [for example] if yesterday I ran an hour and I walked so many steps, [that the app shows] these many kilometers or these many [calories] burnt. I find it super interesting.

P10, aged 39 years, moderate PA level, neutral skill with technology

Interviewees often highlighted the importance of clearly defining goals to help them adhere to PA. Goal setting is a behavior change strategy that fits and can be inspired in SCT and SDT. Suggested goals were mostly quantitative and short term, for example, a number of steps, distance, duration, or speed. Participants also mentioned that these should be slightly more challenging each time:

It is very common in my life to set objectives in the short-term, particularly now, and some more on the long-term, but never much on the long-term... not too much… a period that makes sense to me. Yes, I do this [setting goals] a lot.

P02, aged 50 years, moderate PA level, neutral skill with technology

What I’ve proposed during this process [of trying to be more active] was “I have to do this many daily steps, and each day I will be better... each day I will do a little more”.

P06, aged 50 years, moderate PA level, neutral skill with technology

To me, that I’m a very organized person, it sounds very good because it’s a way of controlling, you control what you are doing [in terms of activity].

P13, aged 47 years, moderate PA level, high skill with technology

##### PA Prescription

Participants suggested having a prescribing tool that would help them plan their PA (key component of goal setting), with details on what they have to do and when to do it. It was also ideated as an activity program that would help them recover their physical fitness and routine after treatment. This seemed to be a way of addressing their barriers of lack of time and lack of detailed PA information:

What I had told you before, having a calendar or creating a schedule [in a physical activity app] would be great.

P01, aged 57 years, low PA level, neutral skill with technology

[…] there is a lot of people who don’t know how to plan. It seems great to have a plan of what you have to do and when you have to do it [physical activity], from Monday to Sunday… It seems perfect.

P10, aged 39 years, moderate PA level, neutral skill with technology

[…] you finish treatment and you have abandoned a little exercising [...] and physically you are not the same. Better [to have] that, a program that tells you how to resume everything, how to come back to training.

P05, aged 43 years, moderate PA level, high skill with technology

Some also suggested including recommendations for other types of exercises, not only physical but also for relaxation, meditation, or nutrition:

[...] in those moments that you are not feeling well, maybe you have to do something of relaxation or meditation for those alternative days [...].

P06, aged 50 years, moderate PA level, neutral skill with technology

##### Positive Reinforcement

Reinforcement is a construct that is widely used in SCT-based interventions. Reinforcements are defined as incentives and rewards to encourage behavior change. Participants reported the importance of receiving such positive reinforcements to acknowledge their efforts:

[...] with positive reinforcement, for example, “look how well you did” and “you have completed your daily objective” or “you have little left to achieve your weekly goal” [...]. So, for me [it would be enough] a simple recognition of “you did well”, [or] a funny and convincing “you’re on your way” [...] It is great [to have this].

P02, aged 50 years, moderate PA level, neutral skill with technology

When you reach your objective that, in some form of message, acknowledges it and incites you to continue, and to propose new challenges.

P06, aged 50 years, moderate PA level, neutral skill with technology

##### Absence of Pressure

Another relevant characteristic to some participants was the absence of pressure, as they did not want to feel pressure toward performing PA. This was particularly evident when talking about app communication. Participants reported not wanting to get negative reactions from the app when they did not reach their goals. This is associated with the characteristics, positive feedback from competence support, and absence of pressure from autonomy support:

I wouldn’t like that it would react [...] like “you have failed”. So, what I mean is that the [virtual] coach [when the user does not comply with an objective] would never react with a negative message. [...] [Instead] it should be “cheer up, I will wait for you tomorrow at 9”.

P10, aged 39 years, moderate PA level, neutral skill with technology

[…] if after the goals are not reached, there is no need to get frustrated.

P13, aged 47 years, moderate PA level, high skill with technology

In addition, participants suggested wanting the *just enough* reminders or notifications from the app:

Well, I think it’s quite interesting, if it doesn’t bombard you too much. Imagine that you don’t feel like it, and there’s this annoying thing telling you [to do it].

P01, aged 57 years, low PA level, neutral skill with technology

That would be very good. [...] A reminder to get a move on the physical matter, [...] to push you to get up. [...] But every day disturbing, no.

P11, aged 69 years, moderate PA level, high skill with technology

##### Simplicity and Ease of Use

Participants wanted a straightforward and easy-to-use app. Some reported having difficulty using complex apps and highlighted the need for a simple, visual, and intuitive interface that was easy to learn and very well explained. Making the system easy to operate can support a sense of competence in users:

Many times I uninstall many apps because they are complex and it takes me time that I don’t want to invest, to learn how to use them. I do not like to spend time to learn it. I prefer something more simple and that later can turn into something more complicated [...].

P02, aged 50 years, moderate PA level, neutral skill with technology

[...] simple, that it would be well explained, that it would be simple and brief [...] and easy to use. That it would be intuitive, very visual, that you could find everything you need or what the app offers, instantaneously.

P02, aged 50 years, moderate PA level, neutral skill with technology

If it [the app] wouldn’t imply more work from me [...]. If you could understand well the different aspects it would help of course… that it would be friendly and useful.

P09, aged 64 years, high PA level, neutral skill with technology

##### Mixed Opinions on Playfulness

Interviewees had varied opinions on playful experiences as a motivational factor. A few recognized the potential positive effects of having game-like experiences, which were thought to bring fun into PA:

Wow, that [having a game-like experience] would be very good.

P11, aged 69 years, moderate PA level, high skill with technology

It should be friendly, useful and fun… otherwise, you get bored.

P09, aged 64 years, high PA level, neutral skill with technology

A variety of gamification elements, including points, rewards, levels, avatars, and competition between users, were discussed during the interviews. Participants suggested the option of exchanging points for real rewards or prizes (eg, a session of yoga, swimming, or pilates) or that rewards could be set by the users themselves, and a few perceived receiving badges (eg, virtual medals) as fun. The implementation of different levels of *experience* seemed to be positively received by the interviewees. Some participants liked the idea of a virtual avatar that they could customize to look like them and to be able to see their activity progress through it. Finally, a few of them also believed that competitions with others could be interesting:

Well, if what you receive, the rewards, can be exchanged for something [...], the pilates session or anything like that...

P14, aged 55 years, low PA level, high skill with technology

And you’ve never thought about rewards being defined by each one? [...] I’d leave it a bit open, you know? [...] [For example,] I will reward myself going to a spa or a weekend getaway [...]

P07, aged 56 years, moderate PA level, high skill with technology

It is kind of a fun game, where I also imagine that you get challenged, right? You will want to level up... it seems good to me.

P03, aged 48 years, moderate PA level, high skill with technology

I like the avatar a lot and also with the dimensions, not a thin avatar. I am now like this, you put me like this, and as we evolve, my little avatar will be losing weight together with me.

P08, aged 47 years, moderate PA level, high skill with technology

But the aspect of competitions with others or doing it with a social network, I think that is quite interesting [...] it always helps you a lot in a challenge or simply to encourage you [...].

P05, aged 43 years, moderate PA level, high skill with technology

However, mixed opinions prevailed with some participants, suggesting that these game-like elements were not motivating for them, as they felt more appropriate to younger people or for those who are into games or are more competitive:

But we are not children. Points do not motivate us.

P04, aged 48 years, moderate PA level, high skill with technology

I think it does have something fun. What I don’t know if all people, depending on age, may like it more or less.

P06, aged 50 years, moderate PA level, neutral skill with technology

There are those who like the theme of games so much, but it is not my case, so I tell you that if they give you four suns or three moons for having achieved it... come on, that does not call my attention at all, I do not find it attractive.

P13, aged 47 years, moderate PA level, high skill with technology

To me, the competitions don’t… they put me nervous, I don’t like them.

P12, aged 66 years, moderate PA level, high skill with technology

It was also suggested that the game experience should be optional to suit every user:

I believe that this part should be optional you know? You can start the game or not start the game … in that way, what happens is that the competitive person is stimulated, but the person that is not competitive and that can have certain stress from this [game], does not have to enter in this with herself.

P07, aged 56 years, moderate PA level, high skill with technology

##### Mixed Opinions on Interacting With Other Users

Some participants were interested in the idea of having a kind of social network in the app that would allow them to have some type of contact with other users. They liked the possibility of sharing, comparing with others, and getting recognition by others. It was also suggested that there could be a common goal for groups of users:

But the aspect of competitions with others or doing it with a social network, I think that is quite interesting […] because you go with other people to do something and the company and sharing it with other people is essential, at least in my case. [...]

P05, aged 43 years, moderate PA level, high skill with technology

I would not mind that it would be connected to Facebook, well it would be a way to upload it to Facebook and say I have improved this much. Some little message that is made public. But yes, yes, the recognition of others is important.

P03, aged 48 years, moderate PA level, high skill with technology

[...] that there would be several people and they could connect [...]. That they would set the goal of this week to walk this much, then to see who achieves it or something like that. That several people are in that group and everyone goes for the common goal.

P14, aged 55 years, low PA level, high skill with technology

Some participants did not feel that such features would suit them and thought it should be optional:

That it exists, that’s fine, there’s people who this would suit, specifically, to me, it would not suit me… I don’t know. [...] we are complicated people, it’s not easy, so… [...] I believe it should give the option in case there is people who benefit from it, but to me personally it would be complicated [...] it repels me.

P09, aged 64 years, high PA level, neutral skill with technology

It was also suggested that it could depend on the current treatment stage:

Well, I think it depends on the moment, right? Because when you are alone when you are in the first period [after treatment] maybe the digital coach will suit you. When you’re a little better, maybe the social network and when you’re pretty good, competition with others.

P06, aged 50 years, moderate PA level, neutral skill with technology

#### Personalization Aspects of the PA Coaching Experience

This theme highlights the participants’ attitudes toward personalization and their perspectives on aspects that should be considered for creating an individualized PA coaching experience: targeting user characteristics, individualized progress information, dynamic adjustment of training, considering the user’s situational context, and interface simulating a virtual coach.

##### Attitudes Toward Personalization

The participants had very positive feelings toward the idea of having an app experience individualized to each user. They often associated tailoring, or personalization, to an increase in user satisfaction or an increase in user engagement. Participants highlighted its particular relevance to address the individual needs of each breast cancer survivor:

Yes, totally, because the more it suits the interests of someone the more satisfied the person will be.

P07, aged 56 years, moderate PA level, high skill with technology

I think the idea that it is something personalized is very important. [...], the fact that it collects a lot of data about you and the circumstances of each one and some symptoms and other stuff like that… it seems to me to be the most important in order to create an app if it is different from what there is now that is not that personalized. [...]

P05, aged 43 years, moderate PA level, high skill with technology

Personalized, because they would be treating me specifically. Every person is unique and also every disease.

P13, aged 47 years, moderate PA level, high skill with technology

Some highlighted its importance, particularly in the early stages of their cancer journey:

During the whole process [of the disease and treatment] it [personalization] seems very important to me. Maybe later, not so much, but in those moments, very much. [...]. [...] during the first moments I think it is fundamental [...].

P06, aged 50 years, moderate PA level, neutral skill with technology

##### Targeting User Characteristics

Participants often expressed the need to have an app tailored to their condition as breast cancer survivors but also on an individual level. Several factors were identified to be considered for personalization, including general characteristics (age, gender, weight), physical and emotional status (eg, limitations, pain, and stress), treatment stage and side effects from treatment and medication, and personal goals:

It would be ideal, because then if it is personalized... if that application knows my limitations, or whatever, or if I can add my limitations... the exercises would aim to meet my needs.

P01, aged 57 years, low PA level, neutral skill with technology

Personalized in terms of the side effects produced by a type of illness and medication. […] Maybe, a degree of pain or a degree of ... or a concept of nutrition or to free you of stress. [...] if there is an adjustment for stress or for pain or for whatever... a little more personalized, that’s fabulous, of course.

P02, aged 50 years, moderate PA level, neutral skill with technology

I imagine it adjusted to each person. I cannot have the same guidelines as the person who has participated previously... there has to be an assessment of everything, the age, what type of specific illness you have had, what you want to achieve with what you are doing. I think that all the factors that should be considered, in order to personalize it a little more.

P08, aged 47 years, moderate PA level, high skill with technology

In addition, the user’s preferences were considered as a factor that could be taken into account by the app. For example, it was suggested that the app could consider the user’s activity preferences in the communication provided or in the activity program:

It’s good because then it reminds you of what [activities] you like. Maybe I’m having a day that I’m feeling down, and it reminds me of what [activity] I really like. I like to be in my garden [...]. [So,] I go to my garden and I start doing this [garden activity] that is good for me.

P07, aged 56 years, moderate PA level, high skill with technology

[For example,] “So, look… running doesn’t suit you. You could walk for one hour and a half […]”.

P08, aged 47 years, moderate PA level, high skill with technology

It was also suggested that some of the app functionalities could be optional to suit one’s preferences:

Always that things [functionalities] are optional, it seems ok to me… if something doesn’t suit me, it doesn’t mean it will not suit another person.

P07, aged 56 years, moderate PA level, high skill with technology

##### Individualized Progress Information

Participants suggested that it would be good to get feedback on their current progress toward goal, particularly when they were close to achieving it, as it would work as an incentive to complete it:

[Talking about a commercial app] It tells you “you are very close to your objective today”... that’s very good because it helps you say “ok, I’m going to do it [the activity] for another little while”.

P02, aged 50 years, moderate PA level, neutral skill with technology

In addition, they wanted to access data on their past activity achievements to understand how they had progressed over time:

Something like that [talking about a game that showed progress in graphs], weekly or every fifteen days, I don’t know, that it gives you progress in various formats. Weekly, monthly, at the end of the year and you see how you overcome obstacles.

P06, aged 50 years, moderate PA level, neutral skill with technology

It's like now if you don’t write down how much you walk every day, in the end, they ask you within a month and you don’t know if in the second week I went to walk every day or one day... so I see it very well, for the aspect of keeping track of your physical activity.

P13, aged 47 years, moderate PA level, high skill with technology

##### Dynamic Adjustment of Training

Participants wanted the app to have activity goals adjusted in difficulty to their PA level and progress. This need for goal adaptation is associated with the competence support characteristics of dynamic difficulty and growth adaptation from SDT. It was also suggested that the app could challenge the user by, for example, increasing the proposed distance or speed when walking. This is associated with the concept of appropriate challenge and characteristics of competence support:

That the person who undertakes them [objectives] finds them easy to achieve and that, as we spoke before, these objectives would vary if you have been accomplishing part of them… that they would be each time more complex and allowing you to overcome yourself.

P02, aged 50 years, moderate PA level, neutral skill with technology

That the application would recommend you or dare you to achieve, I don’t know, for example when walking, [to do] more kilometers or maybe in less time, so that you increase your speed… or something like that. It would be interesting. Especially, because it is true that one gets bored when doing certain things... and you stay a little stuck.

P05, aged 43 years, moderate PA level, high skill with technology

Participants expressed a desire for the training to be adjusted to how the user was feeling and to the treatment stage:

That it can register everything that you are going through in that moment to adjust as much as possible the subject of training, you know? To me, it seems super important. [...] For example, the message of “if you are tired walk every five minutes”.

P05, aged 43 years, moderate PA level, high skill with technology

If you are at the beginning of treatment […] if it warns you with a reminder to tell you “today you have already done enough”. That when you have already recovered it is a reason to encourage you… but that it would be also a reason to stop you or to force you to pause [...].

P06, aged 50 years, moderate PA level, neutral skill with technology

##### Considering the User’s Situational Context

Situational context is information that can be used to characterize the situation of a user (not including the user’s characteristics). Most participants preferred outdoor activities and highlighted the importance of weather conditions as an influencing factor for performing PA:

The weather is important because what I do is walking. If it is very hot or very cold I do not go out [...]. This month has been very cold and I have not gone out.

P01, aged 57 years, low PA level, neutral skill with technology

There was an interest in having an app that would be aware of their location to provide relevant recommendations. It was suggested that it could suggest nearby places to perform an activity, provide alternative routes, and provide alternative indoor exercises for rainy days:

[...] everything about the weather and the location [...] it seems good. That it also informs you about what there is around you, what may interest you… it seems interesting to me.

P03, aged 48 years, moderate PA level, high skill with technology

About routes and getting alternative when maybe the weather is bad or whatever, I find it very interesting. […] I think it is interesting if you can get a training program, maybe for doing at home or something like that.

P05, aged 43 years, moderate PA level, high skill with technology

[...] I always go through the same place, then it can suggest other routes.

P14, aged 55 years, low PA level, high skill with technology

In a different aspect of situational context, some participants were also interested in having their sedentary behavior tracked, to have reminders or warnings to avoid such behavior, and recommendations to move more:

If you get an alarm, like “you've been sitting for two hours I recommend you to move” or something else, yes, it would be good. [...].

P03, aged 48 years, moderate PA level, high skill with technology

##### Interface Simulating a Virtual Coach

Participants seemed to like the idea of having a virtual coach, simulating the interactions with a real trainer. They suggested that it was an interesting and entertaining way of providing more personal feedback and guidance on PA:

I imagine it as a coach in a minicomputer, that would be ideal. […] Yes it would be nice, it would be curious to be able to interact...

P08, aged 47 years, moderate PA level, high skill with technology

[...] also, the personal assistant is entertaining and friendly and it gives you the sensation of personalization.

P09, aged 64 years, high PA level, neutral skill with technology

That seems very interesting to me, I think it’s great because it also makes the application more entertaining, brings it closer to the person, at least to me. [...] Yes... maybe the theme of a virtual coach makes it more enjoyable, no? [...] And obviously it is very good because it’s that, it’s a control and it’s something informative.

P13, aged 47 years, moderate PA level, high skill with technology

It was also suggested that the virtual coach could bring a feeling of trust:

[...] maybe the virtual assistant, or creating a chat, make it like, let’s say, like more human, right? And it would give you, perhaps, more confidence.

P13, aged 47 years, moderate PA level, high skill with technology

#### Technology Trustworthiness

This theme gathers insights from participants on system requirements related to trusting the content and with the use of data collected from the app. The subthemes addressed are system and content credibility and data sharing and privacy.

##### System and Content Credibility

Participants highlighted the importance of trusting the information they are provided with. Some expressed their concern about assessing which information found on the internet was appropriate for them:

There are things that I read that are then generic and are contraindicated for breast cancer issues, so that causes me a lot of insecurity in webs… I would like a serious app where I do not put at risk what is really happening to me. Something that does not put my health at risk.

P03, aged 48 years, moderate PA level, high skill with technology

In addition, some suggested having more trust in information and tools if they were recommended or validated by their health care professionals or backed by exercise specialists. This is associated with the concept of verbal persuasion from SCT:

[...] it would be very interesting to see that there are specialists behind, that is guided by people who know what they propose and who know what they have in hand, like: “we are coaches”, “we are athletes”, “we are sportspeople”. This is very important of course. It’s what gives you security in what you are doing...

P02, aged 50 years, moderate PA level, neutral skill with technology

It was suggested that finding a contact from someone involved in the development of the app or finding that there were other users of the app would make it feel more real:

Well, that’s true, that whenever I enter a page there is always some contact, that there is a telephone, an address because it makes it more real, that, although you are on the web, at any given time you can get in touch with a person, like in a more real way.

P13, aged 47 years, moderate PA level, high skill with technology

[If] you can connect through the page with people that are in the same circumstances [...], it makes it more real.

P13, aged 47 years, moderate PA level, high skill with technology

##### Data Sharing and Privacy

Participants showed a positive attitude toward extensive data collection for the purpose of personalizing the experience as long as their privacy was secured. Some highlighted that they would only do it if the tradeoff between personal data sharing and experience benefits was positive for them:

If the benefit for me is greater than the loss of privacy with data management, well I would do it [share their data].

P06, aged 50 years, moderate PA level, neutral skill with technology

The more data, the more personalized and that seems good to me. What I can get insecure about is giving away that data so [...] the application should be safe, through codes, passwords. Yes, because I think that if it’s personalized it’s much better.

P13, aged 47 years, moderate PA level, high skill with technology

However, they expressed their concerns toward data sharing and access by others. Participants agreed to share data with professionals, whereas preferred it to be optional with other users. In general, they expressed their wish to be able to decide who they share their data with:

With medical personnel, with personnel who I know will handle that data, not with anyone. And besides, if you share these data with a medical purpose of improving the application or to help other people… I don’t know, it seems also important to me when it comes to assessing the loss of intimacy.

P06, aged 50 years, moderate PA level, neutral skill with technology

[...] within the community I would not want to share my data with anyone unless I expressly say that I want to share […]. Within the internet community there are many bad people hidden in a profile like this...

P07, aged 56 years, moderate PA level, high skill with technology

Well, I'd like it to have the option of who can see and who can’t.

P06, aged 50 years, moderate PA level, neutral skill with technology

## Discussion

### Principal Findings

In this study, we conducted semistructured interviews with breast cancer survivors to identify specific needs and considerations regarding various aspects associated with motivation and personalization in PA coaching apps. We identified 5 overarching themes that can guide the design of a future solution: (1) barriers to PA, (2) psychological mediators of PA motivation, (3) needs and suggestions for reinforcing motivation support, (4) personalization aspects of the PA coaching experience, and (5) technology trustworthiness. Important findings from this study include the identification of various determinants of motivation associated with PA adherence: perceived control of behavior and goals, confidence and perceived growth, belief in PA outcomes, and social connectedness. A variety of intervention needs and suggestions for reinforcing motivation were also identified: activity monitoring and goal setting, PA prescription, positive reinforcement, absence of pressure, simplicity and ease of use, and mixed opinions on playfulness and on interacting with other users. Importantly, considerations for personalization of the coaching experience were reported: attitudes toward personalization, targeting user characteristics, individualized progress information, dynamic adjustment of training, considering user’s situational context, and interface simulating a virtual coach. In addition, potential barriers for PA adherence were identified, which helped contextualize some of the motivational and personalization aspects, including physical and emotional challenges, lack of time, and lack of information. Finally, aspects of technology trustworthiness were also highlighted regarding system and content credibility and data sharing and privacy. Overall, survivors believed that a simple and personalized app, which addresses their individual needs and preferences and that provides feedback, guidance, and encouragement for the achievement of progressive PA goals, could be helpful in engaging in a more active lifestyle.

Altogether, these findings can inform the design and may help increase the acceptability and sustained interest of theory-based mHealth PA interventions for breast cancer survivors.

### Comparison With Previous Work

#### Barriers for PA

The findings reported in this study indicate that breast cancer survivors often face barriers to PA participation, which may affect their well-being [16,45]. The 4 barriers described by the participants in this study are somewhat connected and can have a ripple effect, as suggested in the literature [46]. Lack of time and inconvenient exercise schedules are usually reported as one of breast cancer survivors’ bigger barriers to PA [47] that can be a consequence of professional work hours and family caregiving roles [16,45]. Cancer-related fatigue, pain, and weight gain were at the center of physical barriers experienced by the participants and could interfere with the daily functioning of the patients along with their quality of life [16,45,46,48]. Emotional problems (eg, not feeling good or stress) were also reported as a PA barrier, and seem to be a consequence of the psychological burden associated with sticking with the long treatment regimens, which may last for the rest of their lives [16,45,48]. Finally, lack of information was also suggested as a key barrier. When patients with breast cancer cannot access proper information, they can end up following wrong and unguided routines, which may cause frustration or even physical strain and, ultimately, make people give up from continuing with PA [16].

Other barriers reported in the literature that were not identified in our results include access to facilities, aging process, seasonal weather, and disliking exercise [16,45,46,48]. However, some participants in our study did suggest that age and weather aspects should be considered as factors for personalizing the coaching experience.

#### Opinions and Preferences for mHealth PA Interventions

In their study, Phillips et al [14] conducted a mixed-methods study to identify the preferences of breast cancer survivors for mHealth PA intervention features. The themes identified from their interviews were importance of relevance to breast cancer survivors, ease of use, integration with wearable activity trackers, providing a sense of accomplishment, and variability in the desired level of structure and personalization. In their quantitative study, participants revealed a preference for daily and weekly progress feedback, newsfeed, activity challenges, scheduling tools, and motivational and reminder messages. The studies are in line with our study, which also demonstrated an interest of the participants for such intervention components. Integration with wearables was not highlighted in our data, but could be used in combination with a mobile PA coaching app to facilitate activity monitoring and avoid the possible burden of carrying a smartphone during PA. In this sense, the system could leverage the unobtrusive nature of wearables during activity when keeping the richer coaching capabilities from smartphone apps [12].

Before that study, Phillips et al [18] conducted quantitative research through web-based questionnaires to explore breast cancer survivors’ interests and preferences for technology-supported exercise interventions. Their findings are consistent with ours in that the majority of their participants reported that personalized feedback was seen as one of the most helpful technology intervention components. In addition, the least rated components by participants were social networking, group competitions, and the ability to see others’ progress, which relate to our findings about the mixed opinions on interacting with other users.

In the formative development of the Bounce app, a mobile app to increase PA in breast cancer survivors, the research team developed a framework and presented a set of guidelines for the design of behavioral intervention technologies for breast cancer survivors [19]. However, given the focus on reporting intervention design decisions based on the proposed framework, their findings from patient interviews were not detailed in the paper. In addition, they considered a combination of SCT and TTM, whereas our study used the SCT and SDT perspectives. Despite this, their empirical findings seemed to be mostly in line with our results, indicating similar constructs, including reinforcements, helping relationships, self-monitoring, goal setting, verbal persuasion, and mastery experience. In terms of the intervention components, the studies are consistent on the possible inclusion of a scheduling tool; progress monitoring tools, with visuals on current activity (distance and time) and weekly summaries; and a progressive activity program, with incremental levels adjusted to the user’s progress. However, their study simply identified the need for opportunities to connect with other breast cancer survivors in a similar context, whereas in our findings, the social networking aspects did not seem to suit everyone. In addition, owing to the difference in nature of the types of activity considered in the studies, some components were different. For example, the Bounce app was designed to include flexibility and strength exercises, which led to requirements associated with reassuring safety (eg, video demonstrations).

In the most recent paper about the Bounce app [24], gamification techniques such as badges and trophies to reward the users and motivational themes for data visualization were explored. However, with regard to the gamified themes, the authors suggest that they have identified the overall preference of users toward more straightforward representations of their numerical data. This, in some way, is in line with our findings on playfulness, as the variability in opinions about gamification prevailed, with some participants finding its potential to create fun and friendly experiences, although others saw it more appropriate for younger or more competitive people.

Our study complements the findings from previous work with qualitative research on breast cancer survivors’ perspectives on motivational aspects and personalization strategies associated with PA intervention adherence. The findings provide a level of detail across psychological, intervention, and individualization needs for mobile PA coaching that, to the best of our knowledge, was not performed in previous studies on this topic.

Overall, studies including ours demonstrate that breast cancer survivors believe that a mobile tool for PA guidance and coaching can be helpful to adhere to the PA recommendations and are interested in a variety of potential features.

Compared with other intervention modalities, smartphones have a portable size and can use built-in sensors or be paired with external sensors for continuous activity monitoring and feedback. In addition, smartphones’ screen sizes, despite being smaller than tablets or portable computers, also allow for creating rich app experiences to the users [12]. Hence, these devices are believed to be the most suitable for real time PA coaching systems. Despite this, it is cautioned that low technology literacy may be a barrier to adherence in some cancer survivors [13,14] and that apps should be designed to be user friendly and not overly complex. However, survivors are typically older adults, and technology use in this segment of the population is increasing rapidly [49]. Future quantitative research should address the feasibility and efficacy of these intervention modalities and specific features in PA coaching systems for this population and consider a high range of digital literacy levels.

### Recommendations for Research and Practice

The findings presented in this study can be explored to design more motivating and personalized mHealth PA coaching interventions for breast cancer survivors. The insights provided help to understand how to satisfy the psychological needs associated with PA adherence and to increase perceived personal relevance and engagement, which in turn are thought to create value, develop intrinsic motivations, and consequently, lead to sustained adherence. A variety of key recommendations and considerations in this direction are provided in Textboxes 1 and 2.

Our findings suggest that key behavioral determinants of PA adherence in breast cancer survivors can be seen through the lens of SCT and SDT and be used to reason and inform the construction of future interventions. For example, the theme of perceived control of behavior and goals was identified as a motivational determinant associated with PA adherence and is related to the component of autonomy from SDT. Given that SDT posits that intervention characteristics such as increase in sense of ownership, absence of pressure, and self-regulation can support perceived autonomy [31,44], an app could be designed to accomplish these by, for example, personalizing and customizing content to the user, not sending negative feedback to users, and providing opportunities for tracking daily activity. Similarly, to help inform such design decisions, the results section analyses the relationship between the suggested intervention needs (eg, progress feedback, PA prescription, positive reinforcement) and the identified psychological determinants of PA motivation (eg, autonomy, self-efficacy). Finally, matching these insights from the users and theory with, for example, the Coventry, Aberdeen, and London-Refined taxonomy of behavior change techniques [50] can help researchers refine or add new components for the design of an app aimed at increasing PA. Textbox 1 presents a number of considerations regarding the integration of behavior change methods based on such taxonomy and on the positive computing book [44].

Despite the mixed reactions toward more social and gamified functionalities, these may have a critical motivational role for some users and should, therefore, be carefully considered in the design, made optional to the user, or have a secondary role in the app. In particular, based on the participants’ perspectives, it seems worth exploring strategies for connecting with similar users, sharing the experience with loved ones, and getting support from a psycho-oncologist. In addition, the use of game-like mechanisms driven toward an increase in intrinsic motivation (eg, including levels and setting rewards that are valued by users and provide a sense of progress) and that do not make the experience childish or very competitive may contribute to engaging the users.

The variability in opinions regarding certain app characteristics also highlights the need for an individual experience and the importance of personalizing content to the users’ preferences. As suggested by Phillips et al [14], mHealth solutions provide a unique opportunity to create highly personalized interventions for breast cancer survivors in real time. The participants in our study considered the following factors essential for individualization: general characteristics (eg, age, gender, weight), physical status (eg, limitations, fatigue, pain), emotional status (eg, general feelings, stress, and anxiety), treatment stage and side effects, personal goals, preferences, PA level and progress, and weather and location. In addition, participants provided a variety of suggestions for a more individualized experience, which align with the model and strategies for real time personalization in PA coaching systems [12,20]. Associating our findings with these strategies can help design a personalized coaching experience for these individuals. For example, the content of feedback can be explored to achieve user-targeted communication by simply including the user’s name in the messages or by considering the user’s preferences and personal goals to present information specific to the user’s interest (eg, if a user’s main goal is weight management, the app could display more specific feedback information about caloric data). In addition, an app may set a training plan and goals based on the user characteristics (eg, age, baseline PA level, and symptoms) and dynamically adjust these through time according to their progress and how they are feeling (eg, mood, stress, pain). More complex forms of personalization, such as self-learning, may also be explored to select opportune moments for the delivery of activity cues or to adjust training difficulty based on system usage. Suggestions for how an app might incorporate these and other personalization strategies are provided in Textbox 2 on the basis of the model for real time tailoring in PA coaching systems [12,20].

**Key behavior change recommendations for the design and development of mobile health physical activity coaching apps and intervention methods (on the basis of the positive computing book and the Coventry, Aberdeen, and London-Refined taxonomy) for breast cancer survivors.**

Sense of ownership; ability to customize

- App should align with the user’s own value-based goals (eg, a user may want to have a more active lifestyle to lose weight, prevent recurrence of disease, or be more fit to be able to carry on with their roles in work and family) in the communication and training provided
- Allow for a certain degree of customization by the user (eg, goals, frequency, timing and type of activity cues [reminders or notifications], and types of activity)

Clear rationale

- Provide a clear explanation of the reasoning behind the app, coaching, training program, and goals; this may include a summary of the evidence in lay terms and may target the user’s own values and goals

Self-regulation, self-monitoring, goal setting

- Set physical activity (PA)–related short-term goals (eg, on a daily and weekly basis) and encourage users to set long-term goals and expected outcomes that are aligned with user’s own values
- Monitor and present visual information on current activity (eg, steps, calories, pace, distance, and duration), progress toward goals, and progress through time (eg, weekly, monthly, and yearly historic data)
- Consider monitoring sedentary behavior (eg, time spent sitting)
- Provide interpretation of overall progress (eg, weekly and monthly) relevant to user’s values and concerns
- Consider integrating wearables to facilitate activity tracking and real time feedback

Mastering new skills, dynamic difficulty, appropriate challenges

- Have users starting with goals that are fairly comfortable to accomplish and increase goals and training difficulty over time
- Set users with activity challenges to put their fitness level and skill to test
- Adjust training difficulty and challenges to users performance level

Absence of pressure

- Just enough reminders and notifications
- Avoid negative feedback when goals are not reached; instead, cheer up users and encourage them to come back to it another day
- Allow for periods of rest or periods with light training, when needed

Positive reinforcement, verbal persuasion, constructive feedback

- Positive and casual tone of communication from a trusted source (eg, create feedback messages together with professionals)
- Use clear and easy to understand messages
- Acknowledge progress and achievements; possibility to explore rewards based on effort and that are relevant to the user
- Highlight that the sustained PA achievements may bring relevant health benefits

Assist in time management, use follow-up prompts

- Include a tool for planning and scheduling activity (eg, on a weekly basis)
- Include periodic reminders for activity
- Scheduling and reminders should be customizable by the user (eg, allow users to reschedule activities)

Prompt self-talk

- Encourage users to use positive self-talk for motivation to comply with the plan and during activity sessions

Stimulate anticipation of future rewards

- Encourage users to think about the positive outcomes of achieving their PA objectives on their condition as breast cancer survivors (eg, prevention of cancer recurrence and better quality of life) and on their value-based goals (eg, being more fit to play with their children or grandchildren)

Instructed practice

- Provide a prescription of PA with detailed activities, goals, and explaining or demonstrating proper technique
- Provide feedback on user’s performance of the activities
- Address user’s physical limitations

Stress management

- Consider including guidance on how to cope with emotional challenges
- Suggestions for relaxation and meditation exercises could be included
- Encourage connection with a psycho-oncologist

Playfulness

- Gamification elements could be explored for providing fun and engaging experiences, but should be carefully designed, made optional to the user, or have a secondary role in the app; avoid creating a gamified design (eg, systems for points or rewards) that may be perceived as childish or very competitive
- Points, progress bars, levels, and challenges may be considered to provide a sense of progress to the user; customizable avatars may also be explored in this way by, for example, making them evolve physically along with user’s progress
- Explore rewards that may be exchanged by real things and experiences (eg, sessions of yoga or Pilates); consider using rewards that are valued by the user or that can be in some way customized by them
- Optional *healthy* competitions, activity comparisons, or goal sharing within a user community may also be considered in the design; consider user’s personality in terms of competitiveness and openness to social aspects

Provide opportunities for social support

- Optional feature for sharing experience (eg, achievements, progress) with close ones
- Optional networking feature to connect or to share goals with other similar users (eg, buddy system or feature to team up with others to achieve community goals)
- Consider including feature to allow for connection with a counselor (eg, psycho-oncologist) or an exercise trainer for support

High usability

- Make app easy to operate
- Include straightforward and simple content and interface
- Include instructions on how to use the app
- Include explanations on interface specifics (eg, explanation on what a graph represents) and specific action instructions (eg, “Type in your name in the box below”)

Tailoring (or personalization)

- Provide tailored coaching experiences relevant to the user; tailor to the users on a group level, as breast cancer survivors, and on an individual level
- Individualization factors to consider include general characteristics (eg, age, gender, weight), physical status (eg, limitations, fatigue, pain), emotional status (eg, general feelings, stress, and anxiety), treatment stage and side effects, personal goals, preferences, PA level and progress, and weather and location
- Maximize automatic forms of tailoring to reduce need for data input by the user
- Consider the model of real time tailoring in PA coaching apps and the recommendations for personalization provided in Textbox 2

Provide a sense of trust and privacy; verbal persuasion

- Consider including contact details of the people involved in the app development
- Develop based on insights from breast cancer survivors and, together with experts in the field, take an evidence-based approach and validate with health care professionals (eg, oncologists and experts in PA)
- Make these steps explicit to the user
- Inform the user of the total number of breast cancer survivors using the app
- Provide clear and easy to understand information on data security and privacy methods used
- Include transparent and customizable data sharing (eg, with professionals, researchers, other users)

**Key personalization recommendations for the design and development of mobile health physical activity coaching apps and interventions for breast cancer survivors.**

Feedback

- Present the user with information about their own activity data and progress; use straightforward and visual forms for representing data (eg, clear and succinct text, graphs, and progress bars)
- Consider having an optional feature to present feedback in comparison with other similar users (feedback associated with user targeting and interhuman interaction)
- Communication could be represented as a simulation of a virtual coach

User targeting

- Convey that the communication is designed specifically for them (eg, a message in the app could start with “Based on your activity level and preferences…”)
- Include the user’s name in the communication
- Present activity and progress information based on the user’s likes (eg, user may like to see steps and distance but not calories)
- Present activity suggestions, goals, and information based on the user’s characteristics (eg, age, weight, physical activity [PA] level, physical limitations, and likes)
- Present information and tips considering user’s physical limitations

Goal setting

- Dynamically adjust the difficulty of the training plan or goals based on user’s initial PA level, progress, how they are feeling (eg, mood, stress, and pain), and the perceived difficulty
- Suggest resting periods based on user’s progress and how they are feeling

Interhuman interaction

- Explore optional networking or buddy system features joining only similar users based on user characteristics, PA level, and cancer experience (eg, cancer type, time since treatment, and side effects)
- App could include interactions with a real human coach

Adaptation

- Motivate users to achieve their goals and target progress information on the user’s outcome expectations (eg, prevention of cancer recurrence, increased physical capability, weight loss, and increased satisfaction)
- Depending on the stage they are at in their cancer experience, suggest or highlight different features (eg, in the first stages posttreatment it might be important to have support from a counselor, whereas connecting with other users may only be accepted in later stages)
- Adjust type of communication based on user’s confidence levels and skills. For example, if user has low confidence and skills, the app could provide more verbal persuasion and acknowledgement and then adjust it through time based on the user’s growth
- Assess user’s personality profile to define set of features that are relevant to the user; consider this particularly for more social and gamified features (eg, if the personality traits of a user suggest that the user is socially open and competitive then the app would adapt to include components for networking and competing with other users)

Context awareness

- Consider the user’s location to provide suggestions for outdoor activities and alternative routes
- Encourage users to keep doing PA when traveling with suggestions of fitness facilities or outdoor places to do PA
- Encourage users to do PA on bad weather days and provide suggestions for indoor activities
- Adjust goal setting based on user’s working schedules (eg, if a user works full time during the week then set goals for outside working hours or allow to make up for it on the weekends)

Self-learning

- An app may select opportune moments for delivery of activity cues (eg, reminders, notifications) or suggest new training schedules based on system usage (previous interactions of the user with the system)
- Self-learning can be used in combination with goal setting to infer the user’s progress through time and adjust the training difficulty appropriately
- Self-learning strategies may also be used in combination with adaptation to automatically adjust the user’s model based on their stage in the cancer experience or competence skills to provide adapted content or features to the user
- An app may track user’s response to the feedback and recommendations provided by the system, learn their preferences, and adjust the recommendations appropriately

More personalization also means more data being collected from the user. Hence, data sharing and privacy requirements need to be understood and considered in the design of these solutions. All participants indicated a willingness to share their personal data with the app in exchange for a more personalized experience as long as they could choose which data to share and who accesses the data and whether security would be guaranteed. In line with this, it seems necessary that personal data sharing is optional and transparent, and that users trust the app. Technology trustworthiness may be increased by informing users that the system was created together with health care and exercise professionals, and that there are other individuals like them using it. In addition, it is believed that better system credibility may increase the persuasive capability of these PA-promoting apps [51].

Future work exploring the user-centered design of a mobile PA intervention for breast cancer survivors should take into consideration the insights provided in this and related studies, explore how to best integrate the motivational and personalization strategies suggested here, involve the end users in the conceptualization and evaluation processes, and be informed and validated by professionals. In addition, future research should be conducted to assess the impact of these different features and combinations of features on breast cancer survivors’ engagement with these systems.

### Limitations

The results should be interpreted in the context of its limitations. All participants were recruited from the same oncologic clinic, meaning they had received similar care for their cancer. Our sample may be more engaged with the topic owing to their degree of awareness and moderate participation in PA according to the IPAQ-SF. In addition, all the participants were White, Spanish, and posttreatment breast cancer survivors, and most were educated and had access to technology. Despite their awareness of the importance of PA and their moderate IPAQ-SF levels, almost 60% of the participants did not adhere to the recommended levels in the guidelines, and most reported about the lack of detailed information on PA. In addition, although most participants were highly educated and reported high access and usage of technology, some had a neutral self-reported skill with technology or suggested having low digital literacy during the interviews. Future work should generalize to a more diverse sample of breast cancer survivors, considering age, employment status, received cancer care, educational level, digital literacy, country, and race or ethnicity. In addition, it could be argued that less-active participants may need a substantively different intervention approach. Therefore, future studies should analyze such differences and explore the stages of change of the TTM and its constructs, which may provide useful insights in such a direction. Finally, using qualitative research software such as NVivo could have assisted in the thematic analysis and provide more comprehensive insights into the data.

### Conclusions

This work identifies a number of motivational and personalization factors and strategies to be explored in the design of PA coaching systems for breast cancer survivors from the end users’ perspectives. It was grounded in relevant behavior change theories and techniques and the model of real time personalization, which are believed to help create successful interventions. Overall, the findings suggest the need to develop simple, guiding, encouraging, and trustworthy PA apps personalized to breast cancer survivors at both the group and individual levels. This paper opens up new possibilities for the design of PA coaching experiences for these individuals, which may ultimately help sustain technology adherence and increase PA participation.

Future studies should incorporate the perspectives of health care, sports science, and technical professionals as well as further investigate these findings with the involvement of breast cancer survivors in the design, testing, and implementation of PA app prototypes.

## Appendix

Multimedia Appendix 1 Interview guide for the first part of the sessions.

Multimedia Appendix 2 Slideshow presentation of features for the second part of the sessions.

Multimedia Appendix 3 Participants’ access to technology and technology usage.

Multimedia Appendix 4 Supporting quotation for 5 analytical themes and their descriptive subthemes.

## Abbreviations

IPAQ-SF: International Physical Activity Questionnaire-Short Form
mHealth: mobile health
PA: physical activity
SCT: social cognitive theory
SDT: self-determination theory
SWLS: satisfaction with life scale
TTM: transtheoretical model
