# Supplementary material for: Breast Cancer Survivors’ Perspectives on Motivational and Personalization Strategies in Mobile App–Based Physical Activity Coaching Interventions: Qualitative Study
Source: JMIR Mhealth Uhealth. 2020 Sep 21;8(9):e18867. doi: 10.2196/18867 (PMC7536602; doi:10.2196/18867)
Supplement: Multimedia Appendix 1 [file mhealth_v8i9e18867_app1.docx]

**Interview Guide 1^st^ Part – Introductory Questions**

How do you manage your health?

- - What strategies do you use?

How important is PA for you?

- - How do you include physical activity in your daily life? (eg, walking and home chores)

What barriers have you found for the practice of physical activity?

- - What helps you/ would help you to overcome or to mitigate these barriers?

What helps you/would help you to do more physical activity and maintain a routine?

- - What motivates you/would motivate you?
  - How important is the support from your family or others?

Have you ever used a smartphone application?

(If not) Why not? Did you identify any obstacle?

(If yes) Which one/ones? Any related to physical activity?

- - - - Why did you use that app? What features did you like/found useful, and why? What made you/ makes you want to come back and use that app?
      - Which aspects were not useful or you didn’t like, and why?
      - Which obstacles or problems did you find?

What are your thoughts on a mobile application that would guide you and motivate you to do physical activity?

- - Would you want to use an app like this? Why or why not?
  - How do you think it could help?
  - What features would need to be included to be used by you?
  - How would you imagine it being personalized to you? Would that be important? Why or Why not?
