# Supplementary material for: Breast Cancer Survivors’ Perspectives on Motivational and Personalization Strategies in Mobile App–Based Physical Activity Coaching Interventions: Qualitative Study
Source: JMIR Mhealth Uhealth. 2020 Sep 21;8(9):e18867. doi: 10.2196/18867 (PMC7536602; doi:10.2196/18867)
Supplement: Multimedia Appendix 2 [file mhealth_v8i9e18867_app2.pptx]

## Slide 1
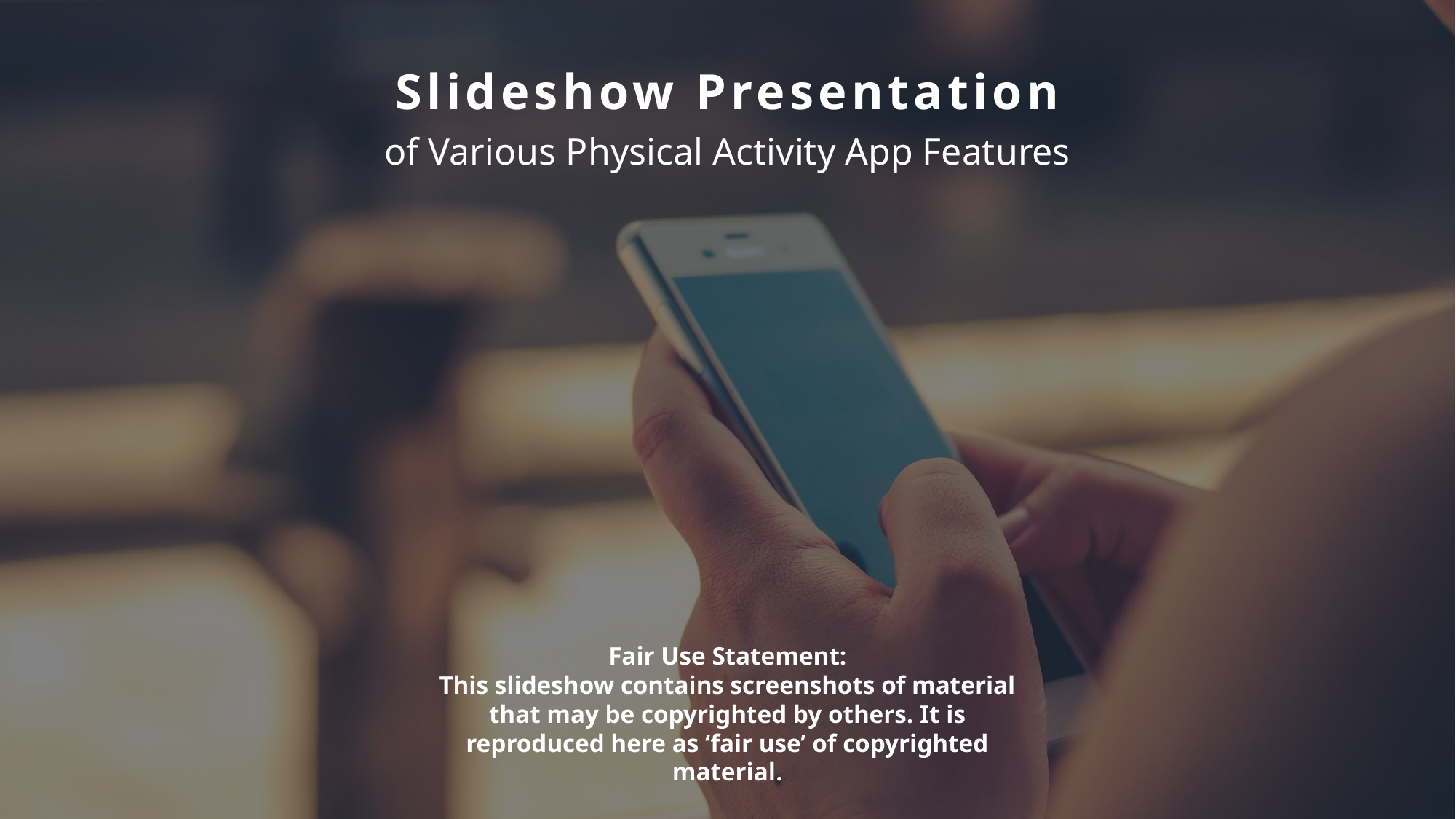

Slideshow Presentation
of Various Physical Activity App Features
Fair Use Statement:
This slideshow contains screenshots of material that may be copyrighted by others. It is reproduced here as ‘fair use’ of copyrighted material.

## Slide 2
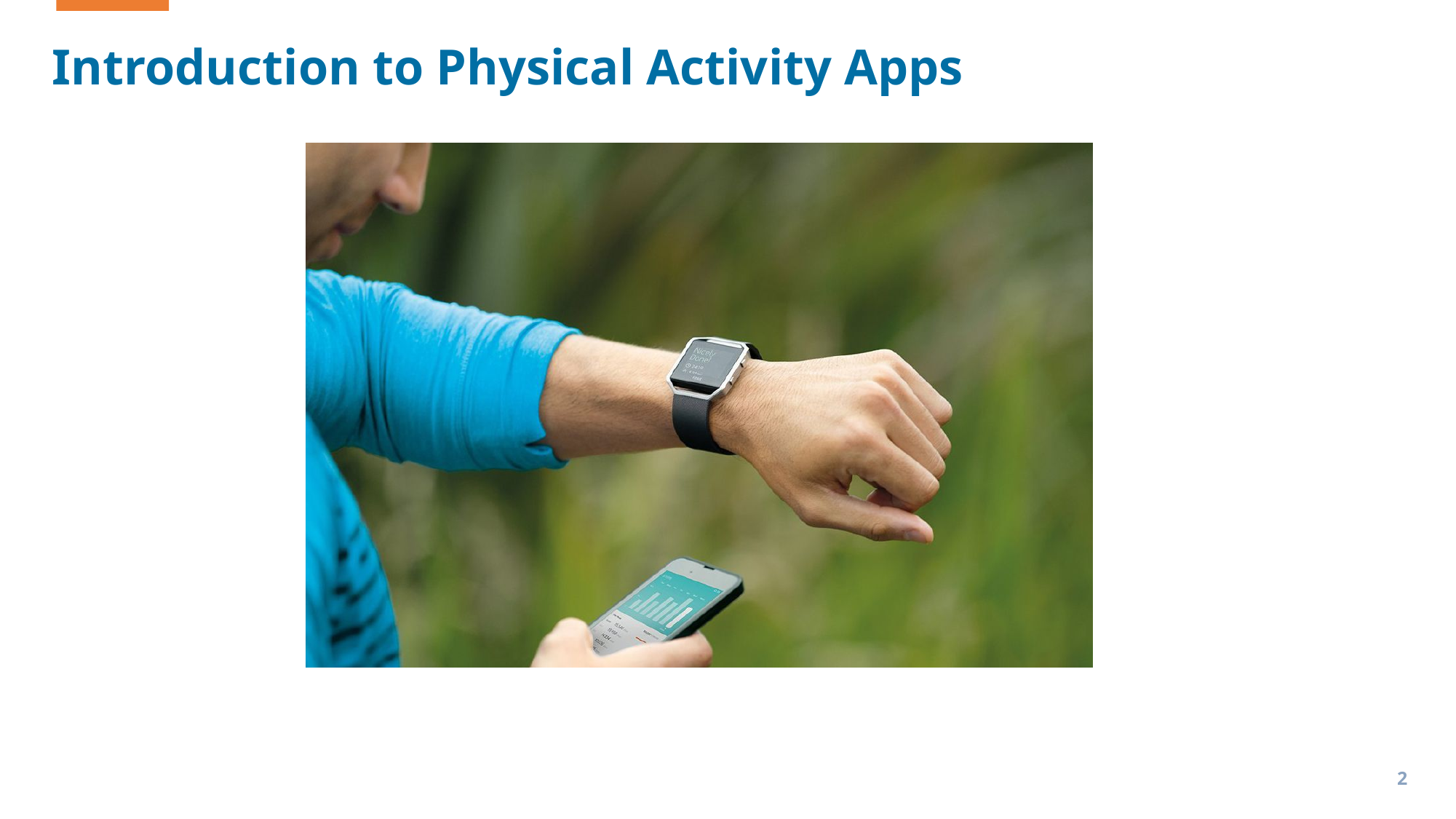

# Introduction to Physical Activity Apps
2

## Slide 3
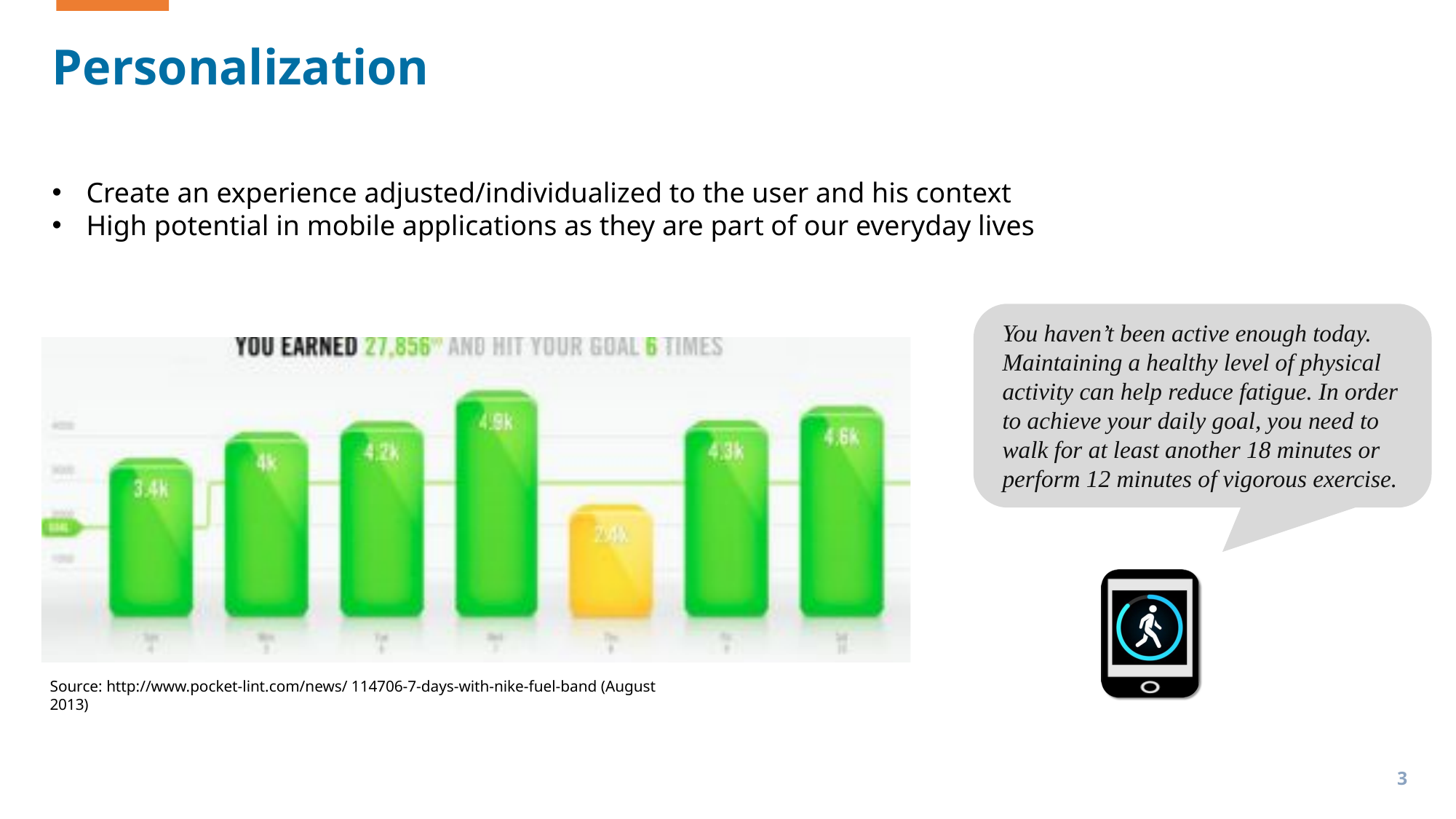

# Personalization
Create an experience adjusted/individualized to the user and his context
High potential in mobile applications as they are part of our everyday lives
You haven’t been active enough today. Maintaining a healthy level of physical activity can help reduce fatigue. In order to achieve your daily goal, you need to walk for at least another 18 minutes or perform 12 minutes of vigorous exercise.
Source: http://www.pocket-lint.com/news/ 114706-7-days-with-nike-fuel-band (August 2013)
3

## Slide 4
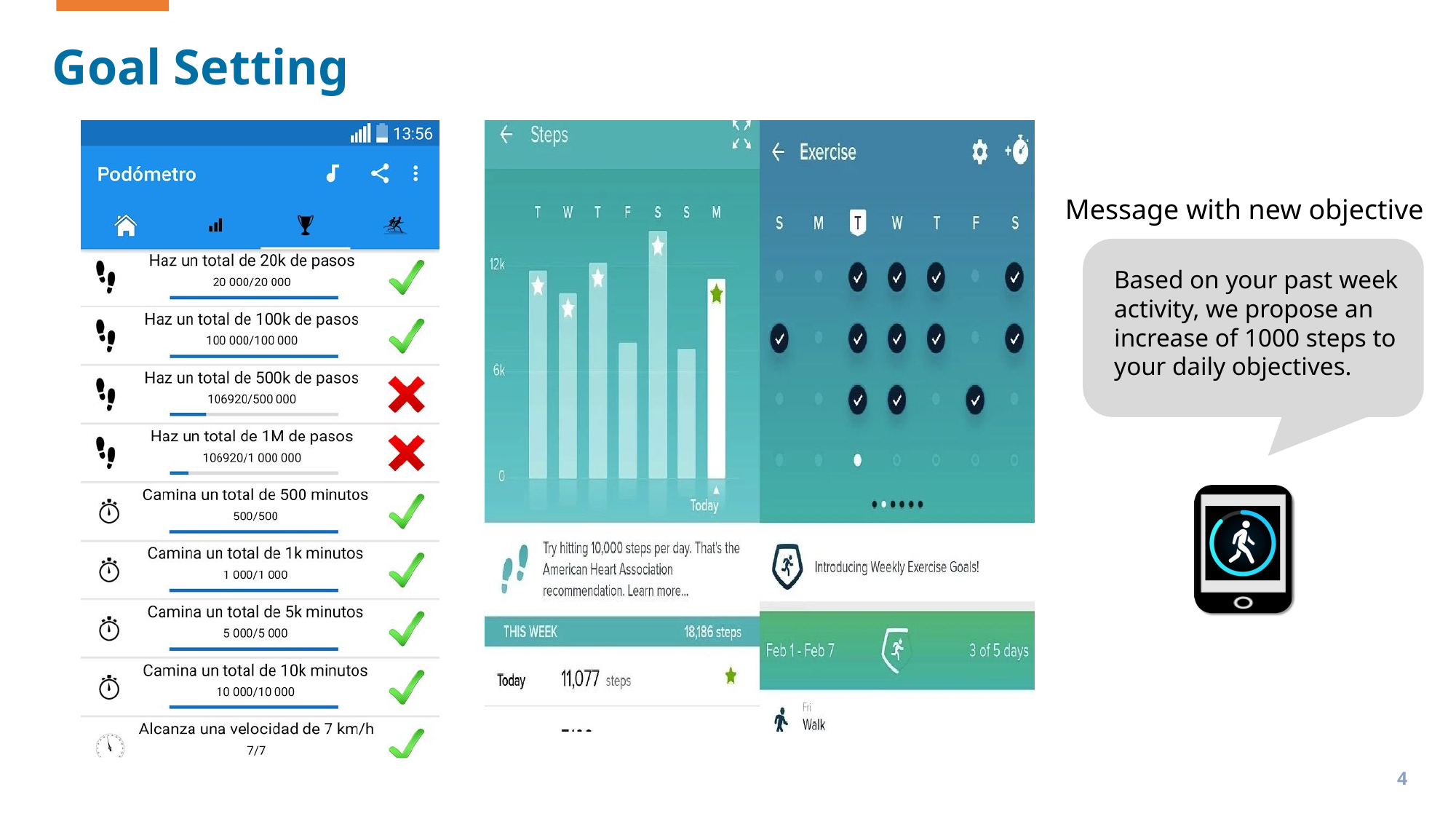

# Goal Setting
Message with new objective
Based on your past week activity, we propose an increase of 1000 steps to your daily objectives.
4

## Slide 5
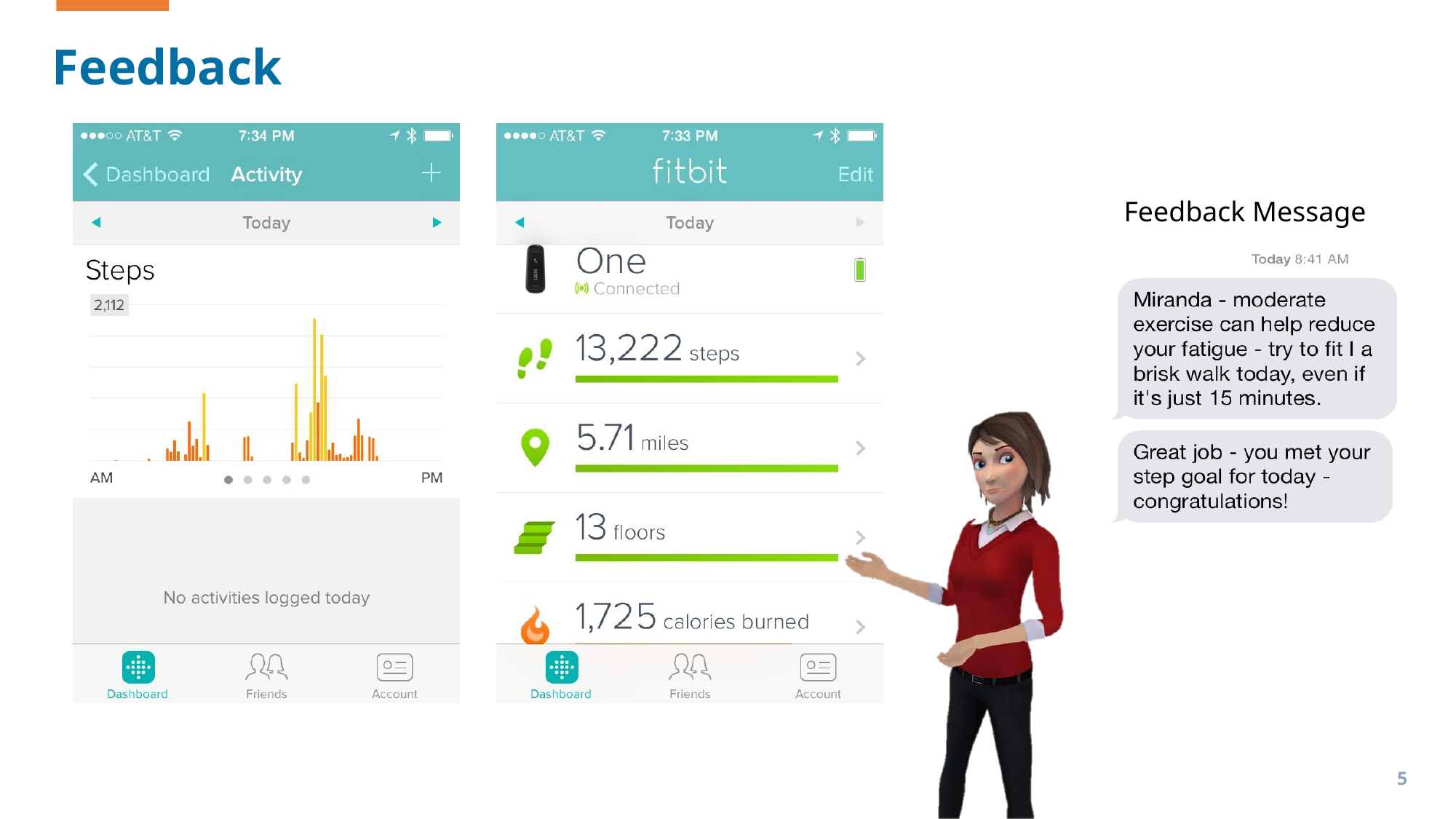

# Feedback
Feedback Message
5

## Slide 6
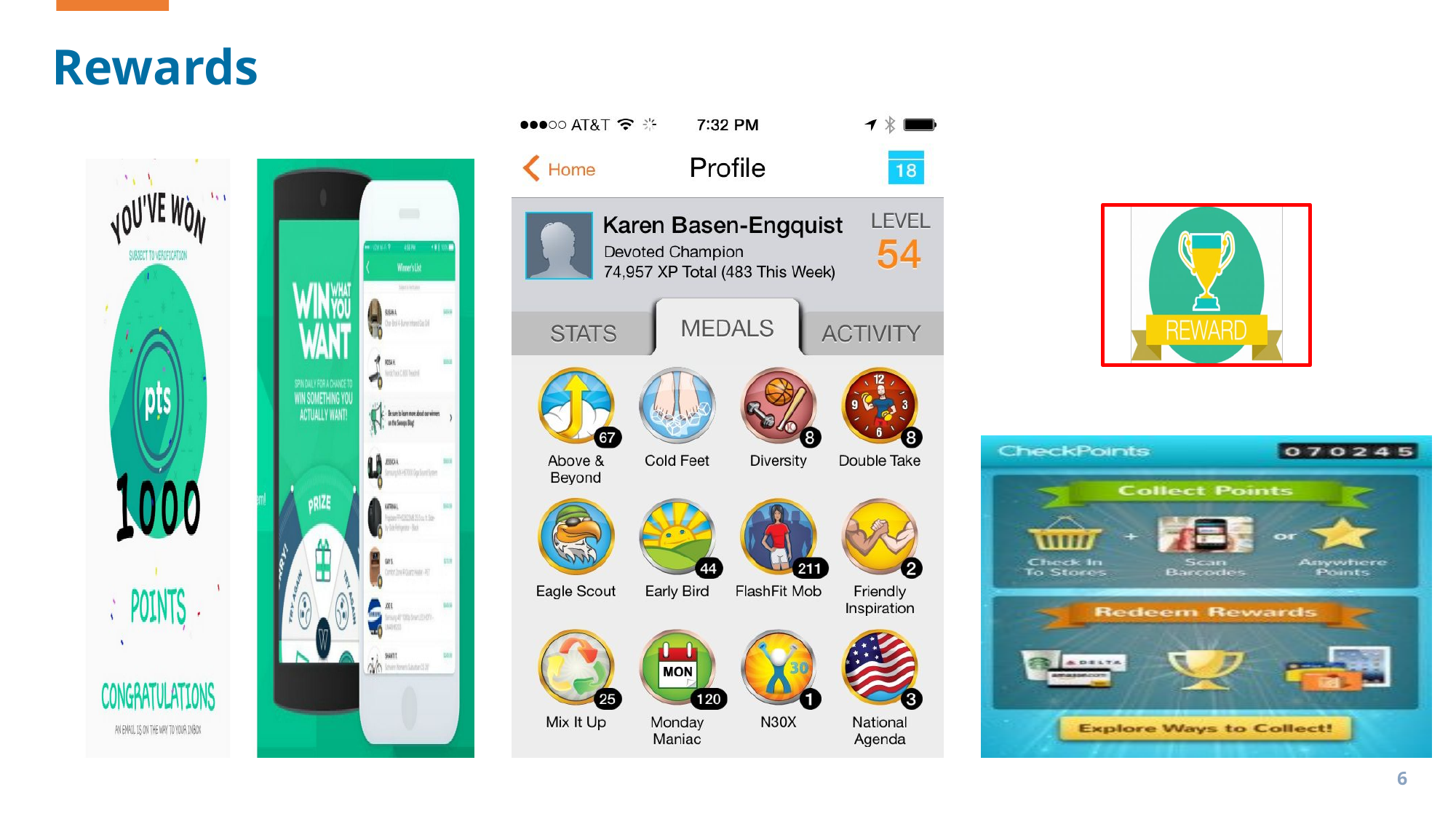

# Rewards
6

## Slide 7
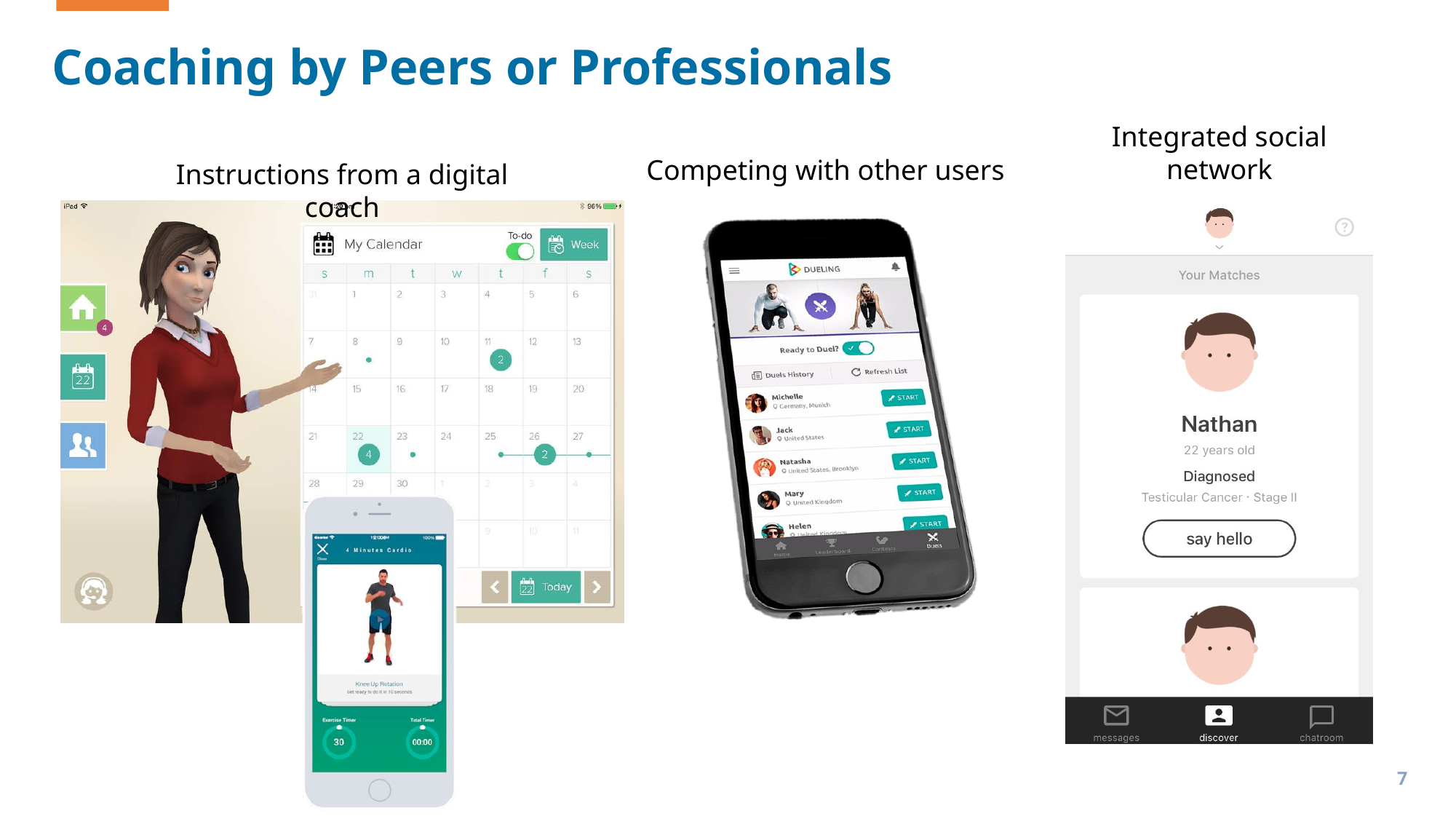

# Coaching by Peers or Professionals
Integrated social network
Competing with other users
Instructions from a digital coach
7

## Slide 8
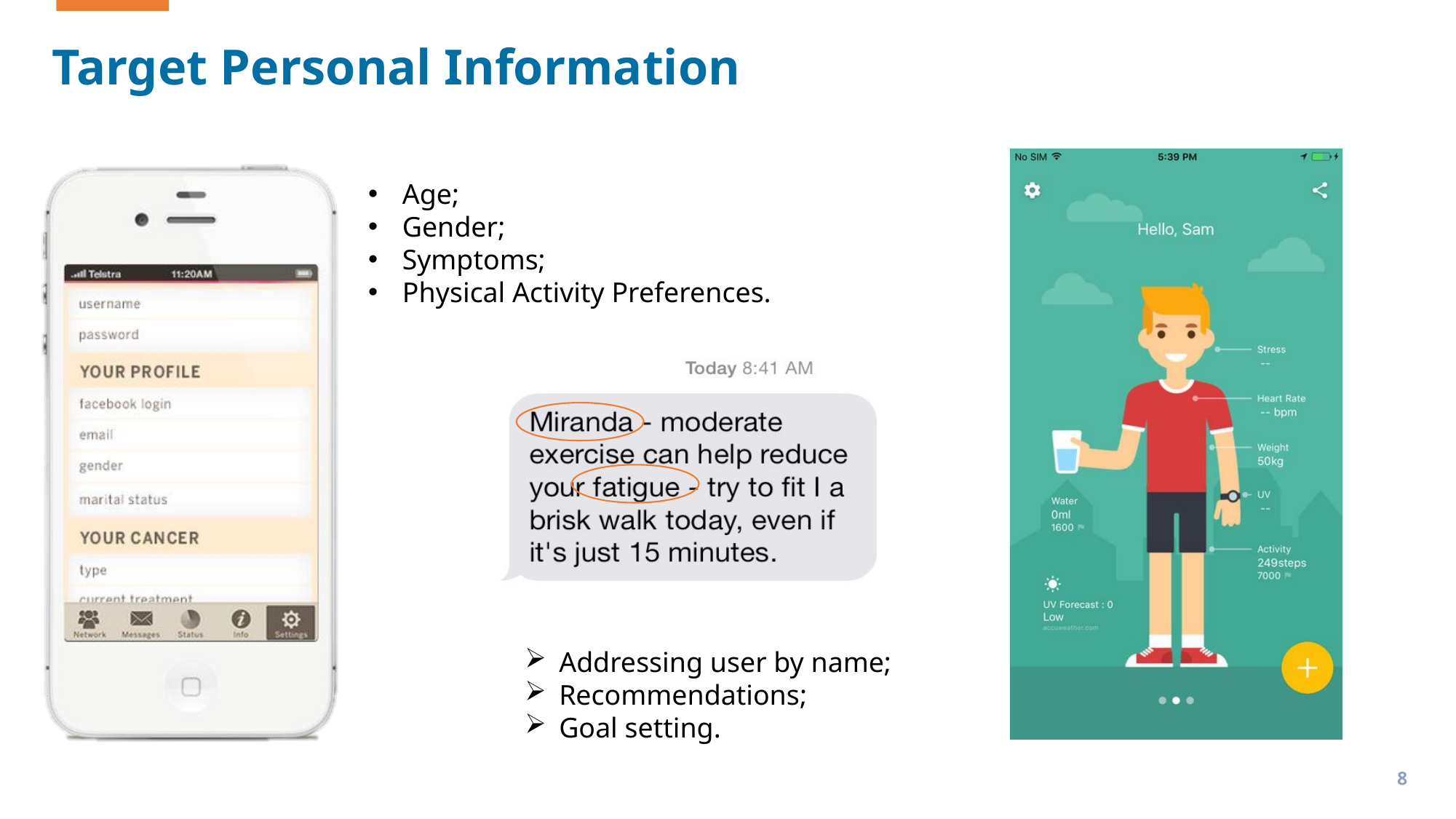

# Target Personal Information
Age;
Gender;
Symptoms;
Physical Activity Preferences.
Addressing user by name;
Recommendations;
Goal setting.
8

## Slide 9
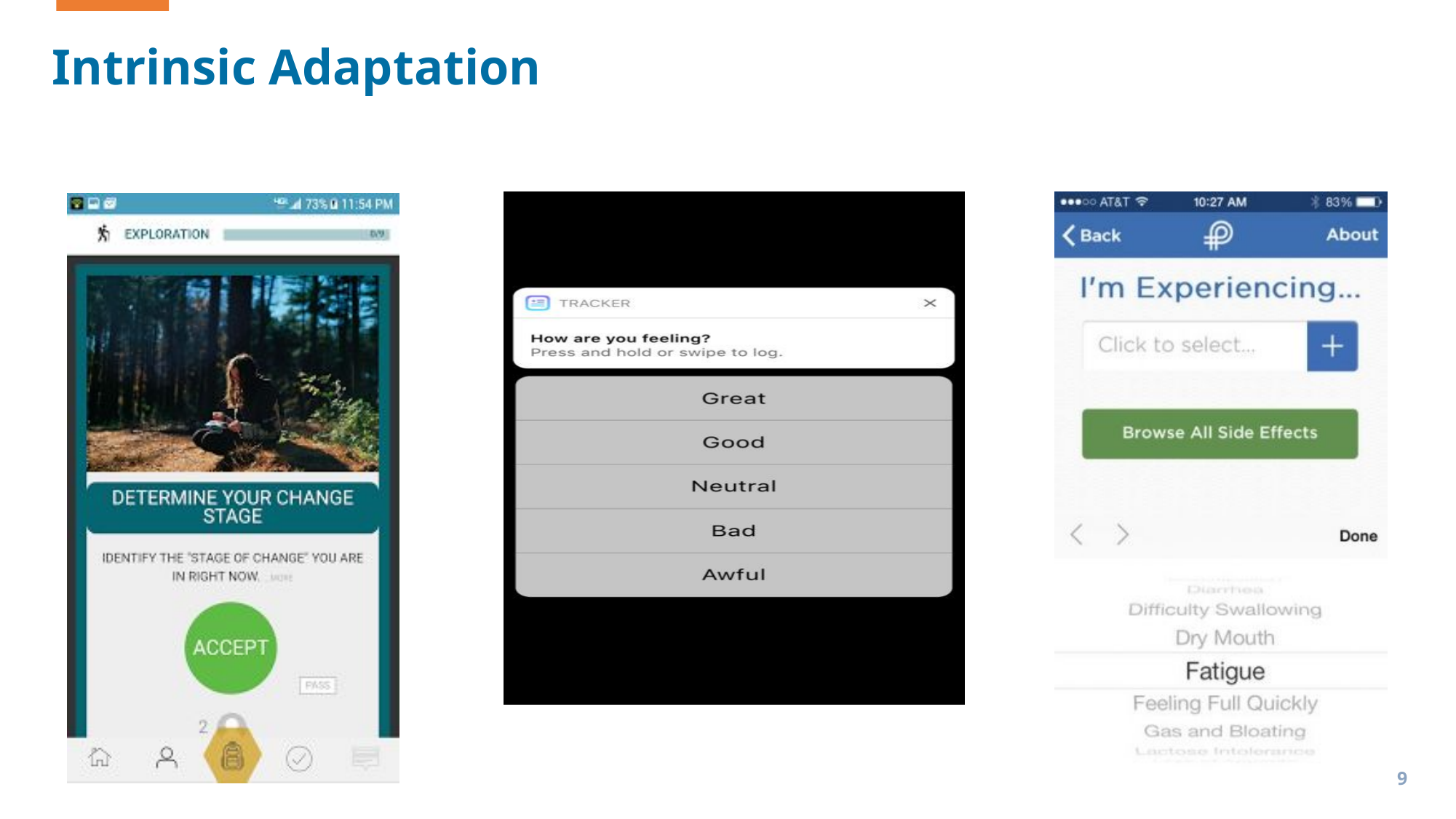

# Intrinsic Adaptation
9

## Slide 10
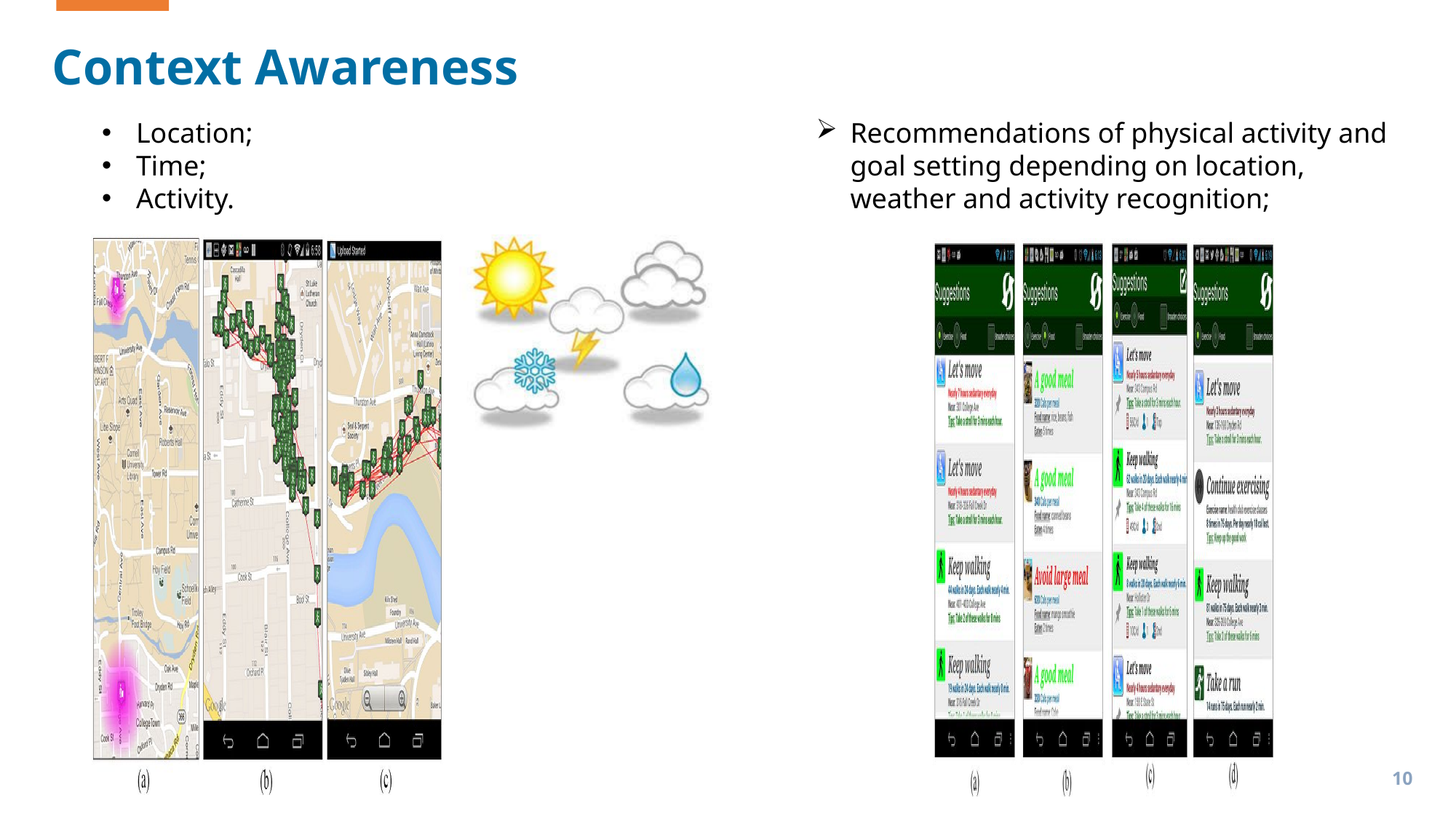

# Context Awareness
Location;
Time;
Activity.
Recommendations of physical activity and goal setting depending on location, weather and activity recognition;
10

## Slide 11
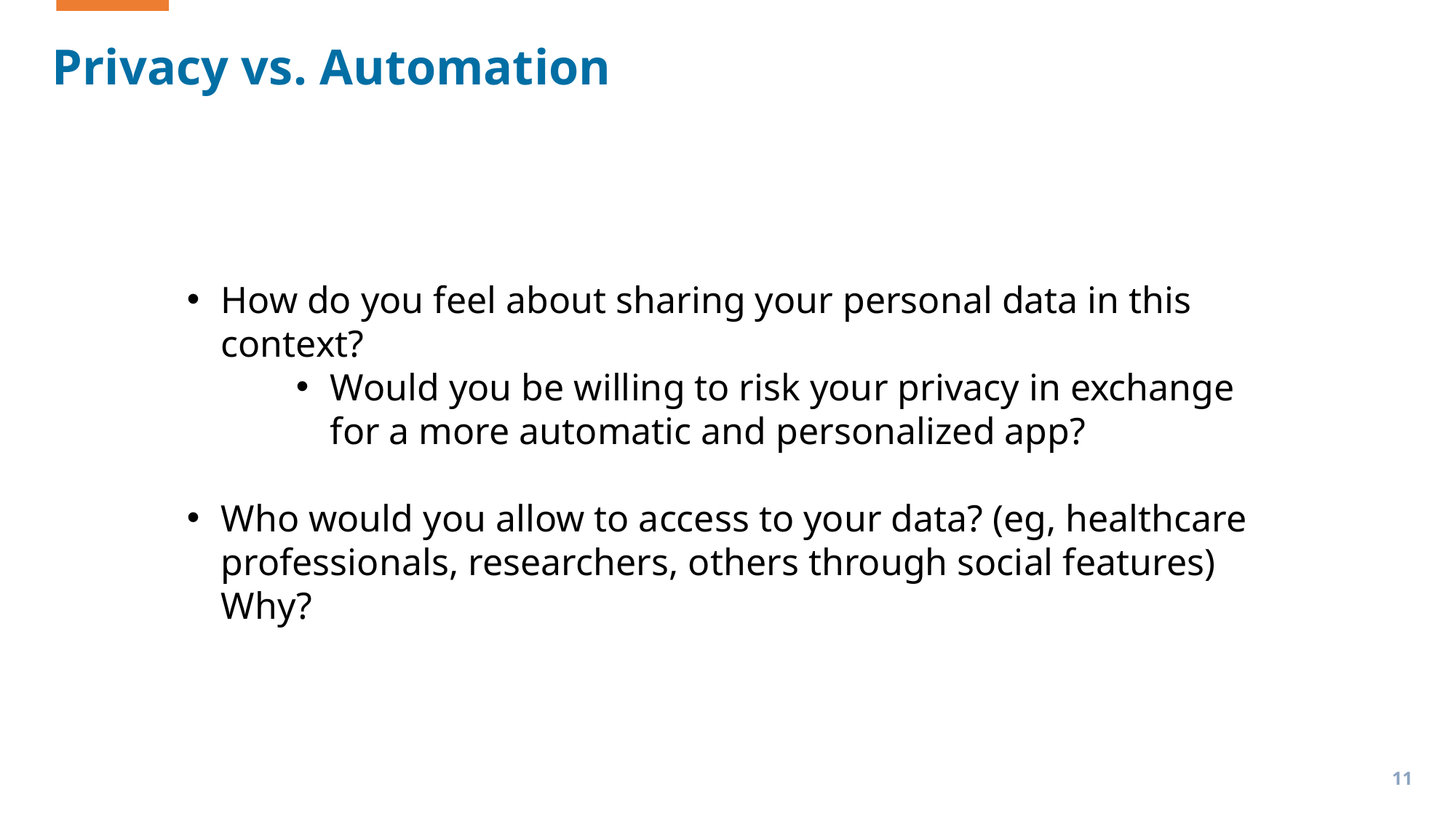

# Privacy vs. Automation
How do you feel about sharing your personal data in this context?
Would you be willing to risk your privacy in exchange for a more automatic and personalized app?
Who would you allow to access to your data? (eg, healthcare professionals, researchers, others through social features) Why?
11

## Slide 12
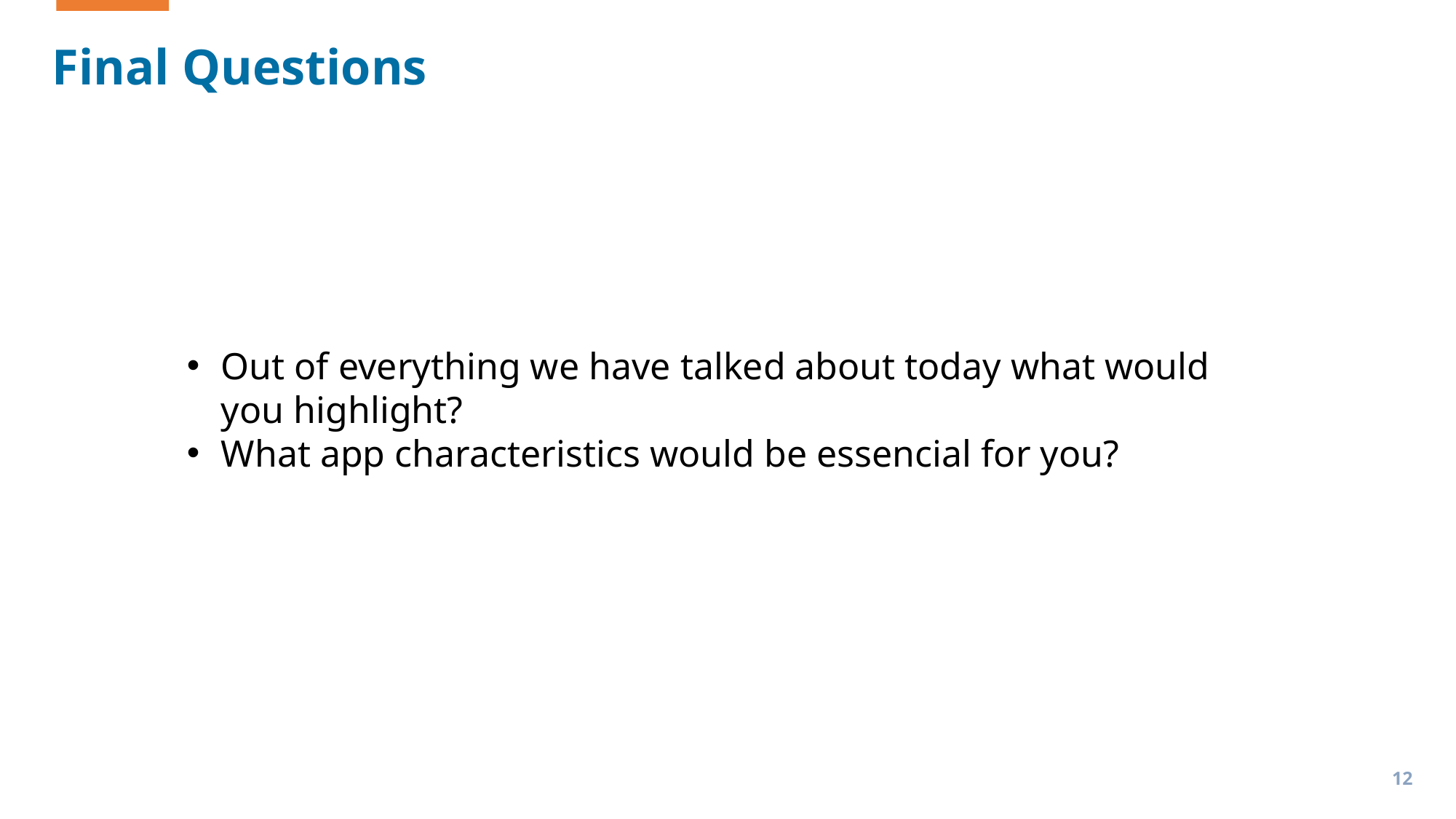

# Final Questions
Out of everything we have talked about today what would you highlight?
What app characteristics would be essencial for you?
12
