# Supplementary material for: Breast Cancer Survivors’ Perspectives on Motivational and Personalization Strategies in Mobile App–Based Physical Activity Coaching Interventions: Qualitative Study
Source: JMIR Mhealth Uhealth. 2020 Sep 21;8(9):e18867. doi: 10.2196/18867 (PMC7536602; doi:10.2196/18867)
Supplement: Multimedia Appendix 3 [file mhealth_v8i9e18867_app3.docx]

Participants’ access to technology and technology usage

**
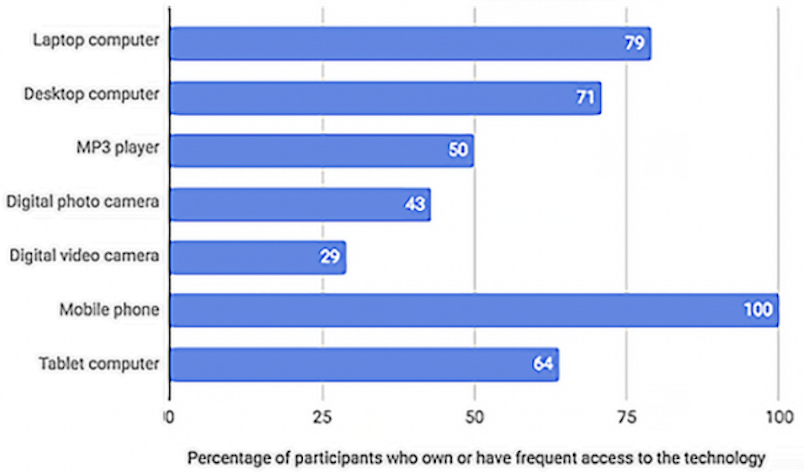
**

Participants’ access to technology (N=14).

**
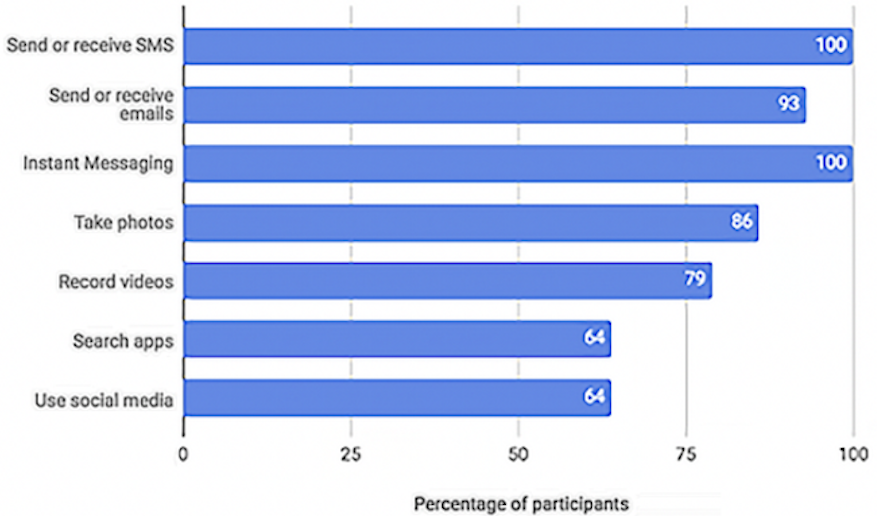
**

Participants’ technology usage (N=14).
