# Supplementary material for: Breast Cancer Survivors’ Perspectives on Motivational and Personalization Strategies in Mobile App–Based Physical Activity Coaching Interventions: Qualitative Study
Source: JMIR Mhealth Uhealth. 2020 Sep 21;8(9):e18867. doi: 10.2196/18867 (PMC7536602; doi:10.2196/18867)
Supplement: Multimedia Appendix 4 [file mhealth_v8i9e18867_app4.docx]

Supporting quotation for 5 analytical themes and their descriptive subthemes

| Analytical themes | | Quotations | |
| --- | --- | --- | --- |
| **1) Barriers for physical activity** | | |  |
|  | Lack of time | *I don’t do much physical activity… It’s very complicated to fit physical activity [in daily routine]. I have a split workday. I start in the morning and I stay until two [o’clock pm], and then I come back at five [o’clock] in the afternoon and I can stay until eight or nine [o’clock pm] in the office, which makes it very difficult [to do physical activity].* [P7]  *We live so fast that we don’t know how to manage time, and [then] we say we don’t have time to do it… [But], when we look for a moment [to do physical activity], then we feel better. The barrier is time management because I’m in an important professional moment. I think that most of us who are young are dedicated to developing ourselves professionally, and on top of that I’m also a mum, so I choose [to leave aside] sports… I leave it [doing sports] as a last priority, which should not be the last priority, I know… But, it is.* [P10]  *The problem is that I struggle to be constant. My daughters are still ten years old, and during the morning I work, I take them to school and I pick them up from school. So, when I don’t have to take them to school, I walk [to work]. But, if I take them [to school], I go by car and I return from work with them by car. At work, I am sitting most of the morning. And, in the afternoon, I combine their extracurricular activities [in own schedule], so I’m not very constant in maintaining physical activity [in everyday life]. It is very hard for me to combine the schedules [hers and her daughters’].* [P3] | |
|  | Physical limitations | *[I felt] many barriers associated with the lymphedema and weight [gained], when doing activity [exercise] with gym equipment. [...] I felt very bad physically, so, even though my head wanted [to do exercise] and people were motivating me [to do it], when it was time to push myself to go [exercise], my body was not responding.* [P5]  *It was hard for me to start [doing physical activity]. [...]. Because I was so fat with the medication [...], fat and without breasts.* [P4]  *It is a need from my body that tells me that I have to move. Because you know that due to the “remains” of the neuropathy, fatigue, tiredness, and muscular pains, either you move or everything [all the physical burdens] will be much worse... it will hurt more or you will be more tired.* [P2]  *I started the first week walking half an hour because, of course, you get out of breath... between what they put in you [referring to medication] and the volume of weight that you gain [...] you get out of breath.* [P8] | |
|  | Emotional challenges | *We are people who stress easily and manage stress even worse now than before the disease. Also because of the medication we receive... it gives some emotional instability…* [P2]  *For a while now I have stopped doing it [physical activity] because I was a little depressed and stressed... that’s why I’ve put on weight.* [P4]  *People start [saying] “Oh, I’m feeling bad, I’m feeling bad, I’m feeling bad...”, and in that way, you don’t recover.* [P4]  *If you are physically unwell, you will also be emotionally unwell…* [P6]  *[One has to] remove the bad thoughts from one’s head and have a healthy mind. [...] The best I can do is to first accept what happened to me [cancer]... and I have already accepted it. [Then,] to be prepared for what may come, because life is tough, and to undo the useless bad thoughts.* [P9] | |
|  | Lack of information | *[...] the order of exercises or of stretching [...] or, maybe, if something hurts, what exercises can be better for you, or what type of things you shouldn’t do.* [P2]  *[...] instructions, maybe, of the best exercises that can be done outdoors and then a set of exercises to do at home.* [P13]  *What makes me angry is that there are many doctors that are still very outdated and the first thing they tell you is to rest… that’s the easiest… and then people ‘rust’.* [P4]  *[...] you often find yourself a bit disoriented [...]. [Talking about mastectomy] I found out much later, once I had already finished the treatment, about lymphedema… I did not know that I had to do some exercises.* [P7] | |
| **2) Psychological mediators of physical activity motivation** | | |  |
|  | Perceived control of behaviour and goals | *It was me [the source of motivation]. Me, because I had to save my children… I was pregnant.* [P4]  *I have been given an opportunity of being here, in life, again... and I have to make the most out of it, doing good for others and being happy.* [P11]  *What I’ve proposed during this process [of trying to be more active] was “I have to do this many daily steps, and each day I will be better... each day I will do a little more”.* [P6]  *[...] Now I’m more interested in the subject of calories, due to the pill that I’m taking, anti-hormone, and all that. I want to have more control over it. [...] Setting goals and objectives is always important. [...] To me, that I’m a very organized person, it sounds very good because it’s a way of controlling, you control what you are doing [in terms of activity].* [P13]  *The objective is to always have some group or activity to sign up to, always… be it pilates or aquagym. [...] not leaving it to when I feel like doing it, but to present myself with a concrete activity. The specific objective is general maintenance, controlling… I do it for my health [...] so that my body doesn’t hurt more [...]* [P1]  *I think that in the end it’s you who sets your own targets and if you feel good with what you are doing then that’s all you need.* [P7] | |
|  | Confidence and perceived growth | *It was hard for me to start [doing physical activity]. Do you know how I started? Going to the spa first. Because I was so fat with the medication…* [P4]  *I felt very bad physically, so, even though my head wanted [to do exercise] and people were motivating me [to do it], when it was time to push myself to go [exercise], my body was not responding.* [P5]  *I went back to training and you want to start almost where you left off, and physically you are not in the same conditions, then it costs you a little, it's like starting from scratch.* [P5]  *I started the first week walking half an hour because, of course, you get out of breath.* [P8]  *In the beginning, when I finished the treatments, it was very difficult [to do physical activity]. But still, I immediately started doing stretching and pilates because the muscles were very damaged. I gained mobility very soon, much sooner than I expected. I had more fear than actual [self-] confidence and I was lucky to go to a psycho-oncologist [...], she encouraged me not to stop doing physical activity.* [P2]  *So, you realize how you are improving, because, of course throughout the time that you are burning calories, you are losing weight and you are improving your performance. [...] You measure your heart rate at rest, and then you do sport and the levels [of heart rate] change... and you have to measure it again at rest another day because the level will increase.* [P4]  *[…] these objectives would vary if you have been accomplishing part of them… that they would be each time more complex and allowing you to overcome yourself. So, in this way, […] you start feeling better because every time you can achieve more objectives, doing more stuff or goals.* [P2]  *[Explaining difficulties with relapse]. I think it motivated me psychologically that I could say “I did it [recovered] and I was capable of doing it”, and it was extremely complicated… [it was] a pretty hard road [process of recovery].* [P5]  *[One has to] remove the bad thoughts from one’s head and have a healthy mind. [...] The best I can do is to first accept what happened to me [cancer]... and I have already accepted it. [Then,] to be prepared for what may come, because life is tough, and to undo the useless bad thoughts.* [P9]  *I get motivated by saying to myself: once the objective is reached, I will feel a greater satisfaction for having achieved it, and it will make it easier for me... whatever way it is... well, in some way in life for sure.* [P2] | |
|  | *Belief in physical activity outcomes* | *If I don't move my knee hurts, my head hurts... and when I exercise I feel better.* [P1]  *The fact that you can move more, helps you relax your mind. The fact that you can find yourself more agile, helps you to feel better about yourself… [...] I believe sport is fundamental [...], it’s about constant improvement… that, for people who come out of cancer [...], and that have been in a capsule of medicines, pain and mental focus [...], when that capsule opens you are so broken that any reasonable target is seven or ten steps that you climb… sport helps a lot in that sense.* [P2]  *It motivates me that I feel better [by being active]… I feel much better physically and psychologically. More lively, as if with more strength, energy… yes, that’s what motivates me.* [P5]  *I have realized that I need certain physical activity because the days that I don’t do it, everything hurts.* [P7]  *It is quite important because I disconnect a lot. It disconnects my mind as if it frees me. I feel better psychologically and physically.* [P13] | |
|  | Social connectedness | *My oncologist [...], in my disease, has been fundamental. [...] he has been putting me in contact with the rest of the professionals.* [P3]  *I am always working with the support of professionals.[...] Since the beginning [...] I’ve worked with a psychologist in therapy [...] always guiding me [...]* [P10]  *The psychologist is very important because you talk to her [...], she cheers you up and then you feel like coming back and do stuff [with the group].* [P5]  *[After treatment] I had more fear than actual [self-] confidence. I was lucky to go to a psycho-oncologist [...] and she encouraged me not to stop doing physical activity… I also met [name of an exercise trainer] [...] which was a very important help.*[P2]  *I started to perform some physical activity to improve my muscle mass, as the physio told me [...]* [P13]  *[...] you have to walk, even when you don’t feel like it. And so I went walking [...], and doing what I think the doctors tell me that is good [for me].* [P13]  *[...] if you get together with someone that has a similar experience, and you talk and share your feelings, that’s a support.* [P1]  *Especially when you are with people who are going through the same as you, then you vent a lot [...] sometimes you don't have to talk, just hanging out, laughing and disconnecting from problems... it helps a lot.* [P5]  *[...] I love talking to people who have gone through the same path as I did.* [P10]  *Especially, because an individual might get bored when she/he does certain tasks and she/he does not progress. Doing PA in a group is different, but if you are doing alone, you do not progress, and sometimes you give up because you get bored. When you do not progress, it is interesting if someone motivates you in some way.* [P5]  *One very good thing about flamenco is that you can share [the activity]. I think that another very funny thing about activities is to be able to socialize with other people.* [P7]  *I like to always go with someone. If maybe she/he does not feel like it, because she/he is doing this and that, then I can join him when he wants to walk.* [P8]  *Seeing how [the trainer] trained other women and see how she made them swim and do exercises… to me, that was important, truly.* [P2]  *I tried not to be involved with other people [...]. When I was sick I have not contacted with other sick people. [...] I have avoided establishing any relationship* [P3]  *[People of the group] were talking about the disease, the types of intervention, etc.: “This doctor is not good” and then it turns out they were talking about your doctor; “This type of intervention no” and it turns out it was yours; “Now [some patient] is undergoing surgery and it seemed awful, awful.” [...] [Name of the oncologist] once told me “don’t interact with anyone... interact with people who don’t have cancer”.* [P3]  *I liked finding a group [talking about a Nordic Walking group], and I really liked Nordic Walking. But, for example, in that case, it was my own [physical] limit [as the barrier]... because I was in one homogeneous group [with higher PA level] and I was asphyxiated [couldn’t handle the activity level].* [P3]  *I try to share these goals with the people around me so that they know [my goals] and they can help me, as a support network.* [P2]  *I do sports with my children... I do horse-riding with my children on the weekend.* [P4]  *Yes, my husband is also a sportsperson and he loves it… and between the two we try to find a free slot [to do activity]. We take turns because we have a child [...]. We find the right moment [to go in turns] or we share it… we both go walking together. The truth is that sports are a very important aspect of our lives.* [P2] | |
| **3) Needs and suggestions for reinforcing motivation-support** | | |  |
|  | Activity monitoring and goal setting | *The steps, the time that I have been exercising and the calories…* [P13]  *All the information [regarding own PA] seems important to me… the more information you have on an exercise that you are doing, the better. Everything seems important because sometimes you are guided, I don’t know, I’m used to be guided more by the distance for example than by steps...but well, everything is important. The more you know about what you are doing the better.* [P5]  *For a diary [referring to the presentation of the daily activity], I find it super motivating… [for example] if yesterday I ran an hour and I walked so many steps, [that the app shows] these many kilometres or these many [calories] burnt. I find it super interesting.* [P10]  *I like it [to get information about activity] [...] knowing how much you’ve walked, how much you’ve run, how much you’ve swum…* [P14]  *It is very common in my life to set objectives in the short-term, particularly now, and some more on the long-term, but never much on the long-term... not too much… a period that makes sense to me. Yes, I do this [setting goals] a lot.* [P2]  *What I’ve proposed during this process [of trying to be more active] was “I have to do this many daily steps, and each day I will be better... each day I will do a little more”.* [P6]  *What happens is that I’ve started with, for example, a walking path of maybe half an hour, and the next day I would try to go more time.* [P12]  *Setting goals and objectives is always important. [...] To me, that I’m a very organized person, it sounds very good because it’s a way of controlling, you control what you are doing [in terms of activity].* [P13] | |
|  | Physical activity prescription | *What I had told you before, having a calendar or creating a schedule [in a physical activity app] would be great.* [P1]  *So, this [having a virtual calendar] I had heard about, and then that it plans it [physical activity] for you... as there is a lot of people who don’t know how to plan. It seems great to have a plan of what you have to do and when you have to do it [physical activity], from Monday to Sunday… It seems perfect.* [P10]  *[...] maybe that it would include some programme more specific for training… because that, it’s true that I don’t know if it exists, but I didn’t find it in these applications. Some programme more specific [...] that it would help you a bit in that, to train… in some way.* [P5]  *Yes, the topic of having a programme that would be for people who are in treatment or that maybe it’s harder for us to resume the physical exercise, because you are a bit out already… because you finish treatment and you have abandoned a little exercising [...] and physically you are not the same. Better that, a programme that tells you how to resume everything, how to come back to training.* [P5]  *Well, look, it could help me perhaps in the order of exercises or stretching or if it had nutrition stuff [...]. Or to relax, relaxation exercises or meditation… breathing too*. [P2]  *[...] that you would include music, meditation.* [P4]  *[...] I don’t know if there is any [application] that considers physical activity and also meditation… because in those moments that you are not feeling well, maybe you have to do something of relaxation or meditation for those alternative days [...].* [P6] | |
|  | Positive reinforcement | *[...] with positive reinforcement, for example, “look how well you did” and “you have completed your daily objective” or “you have little left to achieve your weekly goal” [...]. So, for me [it would be enough] a simple recognition of “you did well”, [or] a funny and convincing “you’re on your way”. [...] Not sure [how the reinforcement should be], but it is great [to have it].* [P2]  *[...] and if it tells you when you overcome a goal, then it cheers you up a little.* [P14]  *When you reach your objective that, in some form of message, acknowledges it and incites you to continue, and to propose new challenges.* [P6] | |
|  | Absence of pressure | *I wouldn’t like that it would react [...] like “you have failed”. So, what I mean is that the [virtual] coach [when the user does not comply with an objective] would never react with a negative message. [...] [Instead] it should be “cheer up, I will wait for you tomorrow at 9”.* [P10]  *[Talking about setting goals]. But, also, it has to be recognized that if after the goals are not reached, there is no need to get frustrated.* [P13]  *For me it would make me feel very stressed, to have this bug [referring to the app] telling me “you did not reach the objective, go out and run for a while”.* [P7]  *I believe that telling you “you have this much left” or “you haven’t done it yet”, sounds that strong… I think that it hurts you a little more. [...] it makes you angry that is says you didn’t get it.* [P8]  *Well, I think it’s quite interesting if it doesn’t bombard too much. Imagine that you don't feel like it, and there’s this annoying thing telling me... but well if it's like that... it’s technology, it’s all about paying attention to it or not.* [P1]  *That would be very good. [...] a reminder to get a move on the physical matter [...] to push you to get up.* [P11]  *But every day disturbing, no… because in the end, it is about turning off the mobile [...]* [P11] | |
|  | Simplicity and ease of use | *[...] I got a daughter that is an expert in social network and I tell her that she needs to help me catch up because there are things that I still don’t know how to use [...].* [P1]  *Many times I uninstall many apps because they are complex and it takes me time that I don’t want to invest, to learn how to use them. I do not like to spend time to learn it. I prefer something more simple and that later can turn into something more complicated [...].* [P2]  *[...] simple, that it would be well explained, that it would be simple and brief [...] and easy to use. That it would be intuitive, very visual, that you could find everything you need or that the app offers instantaneously.* [P2]  *If it wouldn’t imply more work from me [...]. If you could understand well the different aspects it would help of course… that it would be friendly and useful.* [P9] | |
|  | Mixed opinions on playfulness | *Wow, that [having a game-like experience] would be very good.* [P11]  *It should be friendly, useful and fun… otherwise, you get bored.* [P9]  *As a game it’s fun. [...] A bit childish, but that’s ok.* [P9]  *I believe that this part should be optional you know? You can start the game or not start the game … in that way, what happens is that the competitive person is stimulated, but the person that is not competitive and that can have certain stress from this [game], does not have to enter in this with herself.* [P7]  *But we are not children. Points do not motivate us.* [P4]  *I guess to other people that are used to playing games if you give them a prize... For me, it would not be like that, because I am not a person that plays anything or one to feel motivated with application games, in fact they bore me enough, so there would not be something there that would motivate me that much, I think...* [P5]  *There are those who like the theme of games so much, but it is not my case, so I tell you that if they give you four suns or three moons for having achieved it... come on, that does not call my attention at all, I do not find it attractive.* [P13]  *But surely if you find an online store or a sports site, well if you can exchange it for a yoga session, I would like it more for example. “Try this site, you are entitled to a yoga session or an hour of swimming or an hour of pilates”, anything of that kind.* [P2]  *That would be fine. Well, if what you receive, the rewards, can be exchanged for something [...], the Pilates session or anything like that... if it is for something like that, yes. Otherwise, why would you want points? If they are useless, no?* [P14]  *And you’ve never thought about rewards being defined by each one [user]? [...] I reward myself things when I accomplish something that took work so… I’d leave it a bit open, you know? [...] [For example,] I will rewards myself going to a spa or a weekend getaway [...]* [P7]  *Of course, I’d like the virtual medals. The medal collection makes me more excited.* [P2]  *“In this month you have won so many Olympic medals”, that’s good.* [P9]  *It is kind of a fun game, where I also imagine that you get challenged, right? You will want to level up... it seems good to me.* [P3]  *Let's see, if I got the points to level up it would be fine, it would be perfect. And I think the medals and that I think is fine, it is still a game and such, but I think people want the points for something else.* [P8]  *That it has her shape, her style of dressing and everything? [...] Yes, I think it is very entertaining.* [P4]  *It seems fantastic to me, honestly, yes. I like the avatar a lot and also with the dimensions, not a thin avatar. I am now like this, you put me like this, and as we evolve, my little avatar will be losing weight together with me.* [P8]  *Well, I see it much more adapted to young people, this would be liked by my children more, come on, they are twenty-six and twenty-seven years old and I think this would motivate them more. But it is interesting, is not bad.* [P1]  *I think it does have something fun. What I don't know if all people, depending on age, may like it more or less.* [P6]  *But the aspect of competitions with others or doing it with a social network, I think that is quite interesting [...] it always helps you a lot in a challenge or simply to encourage you [...].* [P5]  *The part about competing with others, well it depends on the competitiveness of each one, maybe you are not competitive but the fact that you, this week, have not done it and the other person [...] has lost two kilos, it gives you courage and there is people who get motivated.* [P8]  *To me, the competitions don’t… they put me nervous, I don’t like them.* [P12] | |
|  | Mixed opinions on interacting with other users | *Well, I really liked that it is like a social network, but specific to breast cancer, because there are a lot of associations but no application [...].* [P4]  *But the aspect of competitions with others or doing it with a social network, I think that is quite interesting because whenever you have other people who do the same sport as you, it always helps you a lot in a challenge or simply to encourage you because you go with other people to do something and the company and sharing it with other people is essential, at least in my case. [...] to have people with whom you share that and, above all, I don't know, that thing of “today we are going to do this activity in such a place and competing with other people”, and if not, just sharing even if you don't compete, it seems super interesting to me.* [P5]  *I would not mind that it would be connected to Facebook, well it would be a way to upload it to Facebook and say I have improved this much. Some little message that is made public. But yes, yes, the recognition of others is important.* [P3]  *A training towards a common goal, for example, [...] a race... it occurs to me, the one that there is already every year against cancer, but it could be I don't know, swimming races or whatever. Well, a training for all participants in parallel for all who want to participate in it. Contact between us would be good... like “I'm already here” or “I have already these many points”... and we can train altogether and gather all at the starting point that day and not feel alone in that race, although we don't know each other.* [P2]  *[...] that there would be several people and they could connect [...]. That groups could be formed [...]. That they would set the goal of this week to walk this much, then to see who achieves it or something like that. That several people are in that group and everyone goes for the common goal.* [P14]  *That it exists, that’s fine, there’s people who this would suit, specifically, to me, it would not suit me… I don’t know. [...] we are complicated people, it’s not easy, so… [...] I believe it should give the option in case there is people who benefit from it, but to me personally it would be complicated [...] it repels me.* [P9]  *Well, I think it depends on the moment, right? Because when you are alone when you are in the first period [after treatment] maybe the digital coach will suit you. When you're a little better, maybe the social network and when you're pretty good, competition with others.* [P6] | |
| **4) Personalization aspects of the physical activity coaching experience** | | |  |
|  | Attitudes towards personalization | *It’s true, everything that sounds more personalized is engaging.* [P3]  *Yes, totally, because the more it suits the interests of someone the more satisfied the person will be.* [P7]  *I think the idea that it is something personalized is very important. [...], the fact that it collects a lot of data about you and the circumstances of each one and some symptoms and other stuff like that… it seems to me to be the most important in order to create an app if it is different from what there is now that is not that personalized. [...] It has to be able to register everything that is happening in a specific moment to adequate the training, this is super important to me.* [P5]  *Personalized, because they would be treating me specifically. Every person is unique and also every disease.* [P13]  *During the whole process [of the disease and treatment] it [personalization] seems very important to me. Maybe later, not so much, but in those moments, very much. [...]. [...] during the first moments I think it is fundamental [...].* [P6] | |
|  | Targeting user characteristics | *It would be ideal, because then if it is personalized... if that application knows my limitations, or whatever, or if I can add my limitations... the exercises would aim to meet my needs.* [P1]  *It doesn't have to be so different, but yes adapted to a series of circumstances. I believe that women who suffer from breast cancer, and men too, have a number of, let's say ... side effects that are very similar and I think it's easy to summarize them in the main ones… [that way] it will be easy to guide everyone towards goals. Personalized in terms of the side effects produced by a type of illness and medication. It does not need to be personalized specifically for me, but if at any time there is something that can be more personalized, that would be great, of course  Maybe, a degree of pain or a degree of ... or a concept of nutrition or to free you of stress. [...] if there is an adjustment for stress or for pain or for whatever... a little more personalized, that’s fabulous, of course.* [P2]  *[...] especially that the application can collect as much information as possible, yes, I find it quite interesting. Because age, gender and all that, it is more or less what can be collected by any application... but that it would pick up more specific things from you for training [...]. If it recommends specific things for you according to your side effects, it would be ideal.* [P5]  *I imagine it adjusted to each person. I cannot have the same guidelines as the person who has participated previously... there has to be  an assessment of everything, the age, the specific illness the person has had, what type of specific illness you have had, what you want to achieve with what you are doing. I think that all the factors that should be considered, in order to personalize it a little more.*[P8]  *It’s good because then it reminds you of what [activities] you like. Maybe I’m having a day that I’m feeling down, and it reminds me of what [activity] I really like. I like to be in my garden [...]. [So,] I go to my garden and I start doing this [garden activity] that is good for me.* [P7]  *Some programme more specific, maybe something according to the sport that one does [...].* [P5]  *So, look… running doesn’t suit you. You could walk for one hour and a half [...].* [P8]  *Now I’m more interested in the subject of calories, due to the pill that I’m taking, anti-hormone, and all that. I want to have more control over it.* [P13]  *Always that things [functionalities] are optional, it seems ok to me… if something doesn’t suit me, it doesn’t mean it will not suit another person.* [P7] | |
|  | Individualized progress information | *[Talking about a commercial app] It tells you “you are very close to your objective today”... that’s very good because it helps you say “ok, I’m going to do it [the activity] for another little while”.* [P2]  *[You can see] how many steps you took at a certain point or how much it cost you. Look now, it helped me a lot, I think it's very positive because I think you can see an evolution. If you are constant you will see a real and positive evolution. Many times it is bigger than you expect. You cannot even believe that you have evolved positively so much in a relatively short time [...].* [P2]  *I think it was a little similar to when Nintendo launched a game that was mental training. This game showed your progress and showed you a graph that presents this process. Something like that, weekly or every fifteen days, I don't know, that it gives you progress in various formats. Weekly, monthly, at the end of the year and you see how you overcome obstacles.* [P6]  *It's like now if you don't write down how much you walk every day, in the end, they ask you within a month and you don't know if in the second week I went to walk every day or one day... so I see it very well, for the aspect of keeping track of your physical activity.* [P13] | |
|  | Dynamic adjustment of training | *That the person who undertakes them [objectives] finds them easy to achieve and that, as we spoke before, these objectives would vary if you have been accomplishing part of them… that they would be each time more complex and allowing you to overcome yourself.* [P2]  *That the application would recommend you or dare you to achieve, I don’t know, for example when walking, [to do] more kilometres or maybe in less time, so that you increase your speed… or something like that. It would be interesting. Especially, because it is true that one gets bored when doing certain things... and you stay a little stuck.* [P5]  *That it can register everything that you are going through in that moment to adjust as much as possible the subject of training, you know? To me, it seems super important.* [P5]  *[...] but that it would pick up more specific things from you for training, so, for example, the message of “if you are tired walk every five minutes”...* [P5]  *And I would also find it useful that it would warn you... if you are at the beginning of treatment if you are at the beginning of chemotherapy if it warns you with a reminder to tell you “today you have already done enough”. That when you have already recovered it is a reason to encourage you… but that it would also be a reason to stop you or to force you to pause, [...].* [P6] | |
|  | Considering user’s situational context | *The weather is important because what I do is walking. If it is very hot or very cold I do not go out [...]. This month has been very cold and I have not gone out.* [P1]  *[...] all my life I have played sports and I try in some way or another, to look for some physical activity in winter, I try to dance and in summer to walk or swim or whatever, whatever I need*. [P2]  *It is important. Especially for me, because the physical activity I do is outdoors and for us, it is super important [to know the weather]. Well, if it[the app] recommends new places so we can change our routes, we are always looking for a different park, not to get bored of the same place. It is also important if it [the app] can give you recommendations about training places. Weather recommendations are also important, because of the rain.* [P5]  *[If] I am in Berlin and what I like is to run and walk in natural spaces and next to a museum[...]. I think it's great [those recommendations]... your trip is enriched and it helps you keep up your way of life anywhere. [...] If I am in a congress in Salamanca and someone tells me that there is a park nearby, I'm sure I would take 5 or 10 minutes to go for a walk and eat a sandwich.* [P2]  *[...] everything about the weather and the location [...] it seems good. That it also informs you about what there is around you, what may interest you… it seems interesting to me.* [P3]  *About routes and getting alternative when maybe the weather is bad or whatever, I find it very interesting. Because it is true that sometimes you do nothing when the weather is bad. You do not go for a walk or run or whatever you do. I think it is interesting if you can get a training programme, maybe for doing at home or something like that.* [P5]  *Of course, even if you can't because it's raining then you can go to the gym or whatever and be able to do some kind of activity at home, which can be done perfectly. There are many activities you can do at home.* [P8]  *[...] and the routes also seem fine to me. I always go through the same place, then it can suggest other routes.* [P14]  *Ah perfect, yes, yes. If you get an alarm, like “you've been sitting for two hours I recommend you to move” or something else, yes, it would be good. [...].* [P3]  *That's very good. I had that on the bracelet. When I was a long time [without moving], it sounded and told you to move [...]. I think this is fine.* [P14] | |
|  | Interface simulating a virtual coach | *I imagine it as a coach in a minicomputer, that would be ideal. Because, maybe what I am doing at that moment would not be the most appropriate for the actual state of my health, for my age or for my characteristics, I don’t know. Yes it would be nice, it would be curious to be able to interact...* [P8]  *[...] when you're alone when you are in the first period maybe the digital coach will suit you.* [P6]  *[...] also, the personal assistant is entertaining and friendly and it gives you the sensation of personalization.* [P9]  *That seems very interesting to me, I think it’s great because it also makes the application more entertaining, brings it closer to the person, at least to me. [...] Yes... maybe the theme of a virtual coach makes it more enjoyable, no? ... the application, like more entertaining, no? [...] And obviously it is very good because it’s that, it’s a control and it’s something informative. It is telling you what you have done, the calories, yes it’s good.* [P13]  *[...] maybe the virtual assistant, or creating a chat, make it like, let’s say, like more human, right? And it would give you, perhaps, more confidence.* [P13] | |
| **5) Technology trustworthiness** | | |  |
|  | Finding validation | *There are things that I read that are then generic and are contraindicated for breast cancer issues, so that causes me a lot of insecurity in webs… I would like a serious app where I do not put at risk what is really happening to me. Something that does not put my health at risk.* [P3]  *Well yes, the false messages by WhatsApp, [...] and fallacies and emails or calls to make that are similar to viruses and things like that, I usually try to check before opening anything and on Facebook I have also discovered a lot of information that is false*. [P1]  *[...] if you get on the internet there is a lot of information [...] so, as you are not able to filter it, to know what is good or is bad, I decided not to get in.* [P7]  *[...] I like to be guided by professionals, always. [...] That a psychologist, a nutritionist, a personal trainer participated [in the app].* [P10]  *[...] it would be very interesting to see that there are specialists behind. That is guided by people who know what they propose and who know what they have in hand, like... “we are coaches”, “we are athletes”, “we are sportspeople”... so, the people who know to whom this is directed and that are trained to help you. This is very important of course. It’s what gives you security in what you are doing... you do not run into any kind of risk or any kind of problem added to personal effort.* [P2]  *Well, that’s true, that whenever I enter a page there is always some contact, that there is a telephone, an address because it makes it more real, that, although you are on the web, at any given time you can get in touch with a person, like in a more real way.*[P13]  *Or like a type of forum, yes. Because you can connect through the page with people that are in the same circumstances [...], it makes it more real. You don’t find yourself alone, that you have just entered in a page, but that you know that other people are in it and that you can also communicate.* [P13] | |
|  | Data sharing and privacy | *We already have data everywhere, at least if you are giving it at least that it is something that benefits you, that has a beneficial impact on you. […] If the benefit for me is greater than the loss of privacy with data management, well I would do it.* [P6]  *The more data, the more personalized and that seems good to me. What I can get insecure about is giving away that data so [...] the application should be safe, through codes, passwords. Yes, because I think that if it’s personalized it’s much better. And if it’s an application, it is in principle safe, there should be no problems.* [P13]  *With medical personnel, with personnel who I know will handle that data, not with anyone. And besides, if you share these data with a medical purpose of improving the application or to help other people… I don't know, it seems also important to me when it comes to assessing the loss of intimacy.* [P6]  *With professionals I have no problem sharing them, as long as I have the guarantee that I am dealing with a true medical professional and I am not sharing it with others… This is really a matter of the data protection law.* [P7]  *[...] within the community I would not want to share my data with anyone unless I expressly say that I want to share them […]. Within the internet community there are many bad people hidden in a profile like this...* [P7]  *Well, I'd like it to have the option of who can see and who can't.. As it happens on social networks and everything.* [P6] | |
